# Longitudinal changes in ocular biometry and their effect on intraocular lens power calculation accuracy in cataract patients

**DOI:** 10.1007/s00417-025-06775-z

**Published:** 2025-02-28

**Authors:** Young In Yun, Richul Oh, Joo Youn Oh, Hyuk Jin Choi, Mee Kum Kim, Chang Ho Yoon

**Affiliations:** 1 Department of Ophthalmology, Seoul National University College of Medicine, 103 Daehak-ro, Jongno-gu, Seoul, 03080 Korea; 2 Department of Ophthalmology, Seoul National University Hospital, 101 Daehak-ro, Jongno-gu, Seoul, 03080 Korea; 3 Laboratory of Ocular Regenerative Medicine and Immunology, Biomedical Research Institute, Seoul National University Hospital, 101 Daehak-ro, Jongno-gu, Seoul, 03080 Korea; 4 Department of Ophthalmology, Dongguk University Ilsan Hospital, 27 Dongguk-ro, Ilsandong-gu, Goyang, 10326 Korea; 5 Department of Ophthalmology, Seoul National University Hospital Healthcare System Gangnam Center, 152 Teheran‑ro, Gangnam‑gu, Seoul, 06236 Korea

**Keywords:** Longitudinal change, Ocular biometry, Cataract, Intraocular lens, Calculation

## Abstract

**Purpose:**

To investigate the changes in ocular biometry over time and their impact on intraocular lens (IOL) calculation in adult Korean patients with cataracts.

**Methods:**

Inclusion criteria were patients who underwent two consecutive ocular biometric measurements spaced more than one year apart using the IOLMaster 700 between November 2019 and February 2024 at a tertiary hospital in Seoul, Korea. Longitudinal changes in ocular biometry were evaluated. Predictive errors were compared among patients who underwent cataract surgery using the SRK/T, Kane, Barrett Universal II, Cook K6, EVO, Hill-RBF, Hoffer QST, and Pearl DGS formulas.

**Results:**

A total of 448 eyes from 448 patients were included. Ocular biometry measured over an average interval of 23.4 months showed that with increasing age, axial length elongated (0.04 ± 0.10 mm, *p* < 0.001), and the magnitude of total corneal astigmatism increased (0.04 ± 0.39 D, *p* = 0.018). The mean absolute predictive errors of the final measurements were significantly smaller compared to the initial measurements in the Barrett Universal II, EVO, Kane, and Pearl DGS formulas (difference of -0.05 D, -0.05 D, -0.06 D, and − 0.05 D, respectively). In the subgroup of eyes with an axial length of 25 mm or longer, the final measurements showed even greater reduction in mean absolute predictive errors across multiple formulas, including Barrett Universal II, Cook K6, EVO, Hill-RBF, Hoffer QST, Kane, and Pearl DGS, with reductions of -0.11 D, -0.11 D, -0.10 D, -0.08 D, -0.10 D, -0.09 D and − 0.10 D, respectively.

**Conclusions:**

Axial length increases and corneal curvature changes with aging. IOLMaster 700 ocular biometry results measured closer to the date of surgery were more accurate in IOL power calculation than those measured more than one year earlier, with the greatest improvement observed in myopic eyes. Therefore, it is recommended to repeat IOLMaster 700 biometry before surgery if the previous measurements were taken more than a year ago.

**Supplementary Information:**

The online version contains supplementary material available at 10.1007/s00417-025-06775-z.

## Key messages

*****What is known***:**

- Changes in ocular biometry over time have shown inconsistent results. There is no consensus on the specific timeframe before cataract surgery within which ocular biometry can be reliably trusted.

*****What is new***:**

- Axial length increases and corneal curvature changes with aging.
- IOLMaster 700 ocular biometry results measured closer to the surgery date were more accurate in IOL power calculation than those measured more than one year earlier.
- It is recommended to repeat IOLMaster 700 biometry before surgery if the previous measurements were obtained more than a year ago when using the BUII, EVO, Kane, and Pearl DGS formulas.

**Supplementary Information:**

The online version contains supplementary material available at 10.1007/s00417-025-06775-z.

## Introduction

Cataract surgery is the most common surgical procedure in all medical specialties, with an estimated 20 million cases reported annually worldwide [1, 2]. To achieve accurate postoperative target refraction, it is imperative to obtain precise ocular biometry measurements [3, 4]. IOLMaster 700 (Carl Zeiss Meditec, Jena, Germany), a swept-source optical coherence tomography (SS-OCT) based device, is one of the leading tools used for ocular biometry measurements in cataract surgery [5]. Its longer wavelength light source enhances signal strength and enables better penetration of denser cataracts compared to the partial coherence interferometry-based biometers, thereby improving the accuracy of axial length (AL) measurements [6]. The IOLMaster 700 measures anterior keratometry (AK) via telecentric keratometry. Posterior keratometry (PK) is determined by utilizing SS-OCT technology to capture images of the posterior corneal surface [7]. Additionally, the IOLMaster 700 provides measurements of central corneal thickness (CCT), anterior chamber depth (ACD), lens thickness (LT), white-to-white distance (WTW), angle alpha, and angle kappa. Numerous studies have confirmed the precision and repeatability of the IOLMaster 700 [8–11].

Despite the critical role of ocular biometry in cataract surgery, there is no consensus on the specific timeframe within which measurements can be reliably trusted prior to the surgery. Surgical delays, whether due to patient health issues, such as in elderly individuals, or external factors like hospital closures during events like the COVID-19 pandemic, often necessitate repeat biometric measurements. This redundancy not only increases healthcare costs and patient burden but also places additional strain on outpatient clinic resources. Therefore, a better understanding of how ocular biometric parameters – specifically those measured by IOLMaster 700 – change over time, and how these changes affect IOL power calculations, is essential for determining the clinical necessity of repeat measurements.

Accurately predicting when repeated biometry is required, even in the absence of urgent clinical conditions, is crucial to optimizing surgical planning. Inaccurate IOL power calculations due to outdated or inconsistent biometric data can lead to refractive errors, compromising visual acuity and patient satisfaction. Thus, this study aims to provide evidence-based guidance on the clinical decision-making process regarding repeat biometry assessments, improving both patient outcomes and operational efficiency.

Previous studies on changes in ocular biometry over time have shown inconsistent results [12–15]. Moreover, the impact of these changes on modern IOL calculations and postoperative refractive error has not been explored. Here, we investigated longitudinal changes in ocular biometry over time and assessed their impact on the accuracy of IOL power calculations.

## Methods

This retrospective study analyzed ocular biometry data from the IOLMaster 700 at Seoul National University Hospital (SNUH) from November 1, 2019, to February 1, 2024. This study was conducted in accordance with the Declaration of Helsinki and approved by the institutional review board of SNUH (No. 2112-132−1284). The consent for participation was waived by the institutional review board. The inclusion criteria comprised patients aged 20 years and older with two biometric measurements over a span of one year. The exclusion criteria were as follows: corneal abnormalities, including corneal opacity and keratoconus; retinal diseases that could affect ocular biometric values; intraocular abnormalities; and history of corneal refractive or intraocular surgery. Only successful, high-quality measurements from the IOLMaster 700 were included in this dataset. For patients with both eyes eligible, one eye was randomly selected for inclusion [16]. A total of 448 eyes from 448 patients were included in the study.

Age, sex, AL, AK, PK, total keratometry (TK), anterior corneal astigmatism (ACA), posterior corneal astigmatism (PCA), total corneal astigmatism (TCA), CCT, ACD, LT, WTW, and horizontal and vertical components of angle alpha and angle kappa, were reviewed. AK was calculated by 1000 × (1.3375–1)/R_anterior_, and PK was calculated by 1000 x (–0.04)/R_posterior_. Based on the steep axis of astigmatism, we classified astigmatism into three groups: against-the-rule (ATR) as 0–30º and 150–180º, with-the-rule (WTR) as 60–120º, and oblique from 30–60º and 120–150º. The magnitudes of angle kappa and angle alpha were calculated as the magnitude of the vector, derived from the square root of the sum of squares of the horizontal components and the vertical components.

Longitudinal changes in the ocular biometry values were calculated for all eyes, eyes with initial WTR astigmatism, and eyes with initial ATR astigmatism. Furthermore, changes in ocular biometry values were analyzed separately based on gender, age group (< 50 years and ≥ 50 years), and the presence of underlying conditions such as hypertension or diabetes. Additional subgroup analyses were conducted according to baseline AL (< 23 mm, 23–25 mm, and ≥ 25 mm), the degree of ACA (< 2 D and ≥ 2D), and measurement interval (1–2 years and > 2 years).

Among the 448 eyes, 119 underwent cataract surgery with the Tecnis monofocal 1-piece IOL (model ZCB00, Johnson & Johnson Vision, Santa Ana, CA, USA). We predicted the postoperative refractive errors using both initial and final biometric measurements in eight IOL power calculation formulas: SRK/T, Kane, Barrett Universal II (BUII), Cook K6, Emmetropia Verifying Optical 2.0 (EVO), Hill-Radial Bases Function (Hill-RBF), Hoffer QST, and Pearl DGS. Predicted refraction data using the SRK/T formula were obtained from the IOLMaster 700 (A-constant = 119.30), whereas the other formulas were available via an online calculator (https://iolcalculator.escrs.org/ accessed on June 7, 2024). The predicted error was defined as the difference between the predicted and measured postoperative refractive errors. Mean predicted error (ME), median predicted error (MedE), absolute predicted error (MAE), and absolute median predicted error (MedAE) from the initial and final biometric measurements were compared for each formula. Additionally, ME and MAE were compared between the two biometric measurements for each formula stratified by initial AL (AL: < 23 mm, 23–25 mm, and ≥ 25 mm). In all 119 eyes that underwent surgery, the final biometry measurement was taken within one month prior to the surgery date. Double-angle plots were created to evaluate changes in the average magnitude and angle of the ACA, PCA, and TCA over time. To combine the results of the right and left eyes, the left-eye data were transformed by mirroring the vectors on the y-axis to neutralize the enantiomorphism of corneal astigmatism.

Statistical analysis was performed using the Prism software version 9.3.1 (GraphPad Prism, Inc., San Diego, CA, USA), and double-angle plots were generated using SigmaPlot software version 12.5 (Systat Software, Inc., San Jose, CA, USA). A paired *t*-test was applied to compare the initial and final measurements if the data followed a normal distribution, as determined by the Kolmogorov-Smirnov test. Otherwise, the Wilcoxon signed rank test was used. All *p* values were two-sided, and a *p-*value < 0.05 was considered statistically significant.

## Results

A total of 448 eyes from 448 patients were included in the study, comprising 287 women (64%) and 161 men (36%), with 206 (46%) right eyes and 242 (54%) left eyes. The mean ± SD age at the initial measurements was 64.4 ± 13.3 years (range 20–91). The average time between biometry measurements was 23.4 ± 9.2 months (range 12–51). The mean ocular biometric values are shown in Table 1.

**Table 1:** Longitudinal change in ocular biometry measured by IOL Master 700 in 448 eyes

Parameter	Initial measurement	Final measurement	Difference (Final-Initial)	*p*-value
Age (year)	64.42 ± 13.26	66.36 ± 13.26	1.95 ± 0.77	**< 0.001**^a*^
Sex (male/female)	161 (36%) / 287 (64%)			
Laterality (right/left)	206 (46%) / 242 (54%)			
AL (mm)	24.58 ± 2.04	24.62 ± 2.07	0.04 ± 0.10	**< 0.001**^a*^
Flat AK (D)	43.48 ± 1.55	43.47 ± 1.56	−0.01 ± 0.28	0.129^a^
Steep AK (D)	44.44 ± 1.55	44.44 ± 1.55	0.00 ± 0.30	0.885^a^
Mean AK (D)	43.96 ± 1.51	43.96 ± 1.52	0.00 ± 0.23	0.525^a^
Flat PK (D)	−5.77 ± 0.24	−5.77 ± 0.24	0.00 ± 0.07	0.950^b^
Steep PK (D)	−6.09 ± 0.26	−6.09 ± 0.26	0.00 ± 0.08	0.474^a^
Mean PK (D)	−5.93 ± 0.24	−5.93 ± 0.24	0.00 ± 0.06	0.401^b^
Flat TK (D)	43.45 ± 1.56	43.43 ± 1.57	−0.02 ± 0.33	**0.028**^a*^
Steep TK (D)	44.44 ± 1.54	44.45 ± 1.55	0.02 ± 0.32	0.311^a^
Mean TK (D)	43.94 ± 1.51	43.94 ± 1.52	0.00 ± 0.26	0.817^b^
TK-AK (D)	−0.02 ± 0.11	−0.01 ± 0.11	0.00 ± 0.06	0.514^b^
ACA (D)	0.96 ± 0.65	0.98 ± 0.67	0.02 ± 0.35	0.275^a^
PCA (D)	−0.32 ± 0.14	−0.32 ± 0.15	0.00 ± 0.10	0.350^b^
TCA (D)	0.99 ± 0.66	1.03 ± 0.69	0.04 ± 0.39	**0.018**^a*^
CCT (mm)	0.54 ± 0.03	0.54 ± 0.03	0.00 ± 0.01	0.173^b^
ACD (mm)	3.13 ± 0.43	3.14 ± 0.44	0.00 ± 0.10	0.726^a^
LT (mm)	4.46 ± 0.48	4.45 ± 0.50	−0.01 ± 0.35	0.078^a^
WTW(mm)	11.78 ± 0.43	11.79 ± 0.45	0.01 ± 0.31	0.346^a^
Angle alpha (mm)	0.44 ± 0.22	0.45 ± 0.25	0.01 ± 0.24	0.764^a^
Angle kappa (mm)	0.26 ± 0.15	0.26 ± 0.15	0.01 ± 0.13	0.696^a^

Data are presented as mean ± standard deviation or as number (percent)

Statistically significant values (*p* < 0.05) are shown in asterisk (^*^)

^a^ Wilcoxon signed rank test

^b^ Paired t-test

*AL* axial length, *AK* anterior keratometry, *D* diopter, *PK* posterior keratometry, *TK* total keratometry, *ACA* anterior corneal astigmatism, *PCA* posterior corneal astigmatism, *TCA* total corneal astigmatism, *CCT* central corneal thickness, *ACD* anterior chamber depth, *LT* lens thickness, *WTW* white-to-white distance

During an average interval of 23.4 months, AL increased over time (0.04 ± 0.10 mm, *p* < 0.001). TK of flat axes decreased (–0.02 ± 0.33 D, *p* = 0.028), while flat AK and flat PK showed no significant change. Steep AK, steep PK, steep TK, mean AK, mean PK, mean TK, and TK-AK did not change significantly over time. Magnitude of TCA increased over the same period (0.04 ± 0.39 D, *p* = 0.018). The ACA, PCA, LT, WTW, angle alpha, and angle alpha did not show significant changes. To obtain a detailed look at the change in astigmatism over time, subgroup analyses of biometric value changes were performed with respect to the initial anterior corneal astigmatism. In a subgroup analysis of 231 eyes with WTR astigmatism, AL increased significantly over the period of 23.3 months (0.05 ± 0.12 mm, *p* < 0.001). Flat TK also decreased (–0.04 ± 0.29 D, *p* = 0.009) with aging. (Table 2). In 147 eyes with ATR astigmatism, AL was similarly elongated during the average interval of 23.4 months (0.02 ± 0.04 mm, *p* < 0.001). Steep AK and steep TK increased (0.06 ± 0.26 D and 0.07 ± 0.29 D, respectively; all *p* = 0.005), leading to an increase in ACA and TCA, although the change in TCA did not reach statistical significance (0.06 ± 0.35 D, *p* = 0.026 and 0.06 ± 0.38 D, *p* = 0.054, respectively) (Table 3). Longitudinal changes in ocular biometry values are detailed in Supplementary Tables 1–7, stratified by gender, age group (< 50 years and ≥ 50 years), the presence of underlying conditions such as hypertension or diabetes, baseline AL (< 23 mm, 23–25 mm, and ≥ 25 mm), the degree of ACA (< 2 D and ≥ 2D), and measurement interval (1–2 years and > 2 years). Notably, the magnitude of AL elongation was greater in myopic eyes (0.07 mm, *p* < 0.001) compared to eyes with short (0.03 mm, *p* < 0.001) or normal axial lengths (0.02 mm, *p* < 0.001).

**Table 2:** Longitudinal change in ocular biometry measured by IOL Master 700 in 231 eyes with with-the-rule astigmatism

Parameter	Initial measurement	Final measurement	Difference (Final-Initial)	*p*-value
Age (year)	59.73 ± 14.26	61.67 ± 14.24	1.94 ± 0.79	**< 0.001**^a*^
Sex (male/female)	75 (32%) / 156 (68%)			
Laterality (right/left)	100 (43%) / 131 (57%)			
AL (mm)	25.02 ± 2.29	25.06 ± 2.33	0.05 ± 0.12	**< 0.001**^a*^
Flat AK (D)	43.48 ± 1.58	43.47 ± 1.59	−0.02 ± 0.25	0.177^a^
Steep AK (D)	44.54 ± 1.61	44.49 ± 1.62	−0.04 ± 0.33	0.343^b^
Mean AK (D)	44.01 ± 1.55	43.98 ± 1.56	−0.03 ± 0.23	0.052^b^
Flat PK (D)	−5.73 ± 0.25	−5.73 ± 0.25	0.00 ± 0.07	0.680^b^
Steep PK (D)	−6.12 ± 0.27	−6.12 ± 0.28	0.01 ± 0.09	0.288^a^
Mean PK (D)	−5.93 ± 0.25	−5.92 ± 0.25	0.00 ± 0.06	0.305^a^
Flat TK (D)	43.55 ± 1.57	43.51 ± 1.58	−0.04 ± 0.29	**0.009**^a*^
Steep TK (D)	44.45 ± 1.59	44.43 ± 1.60	−0.02 ± 0.34	0.379^b^
Mean TK (D)	44.00 ± 1.54	43.97 ± 1.55	−0.03 ± 0.25	0.068^b^
TK-AK (D)	−0.01 ± 0.11	−0.01 ± 0.12	0.00 ± 0.06	0.786^b^
ACA (D)	1.05 ± 0.74	1.03 ± 0.75	−0.03 ± 0.36	0.238^a^
PCA (D)	−0.39 ± 0.13	−0.38 ± 0.14	0.01 ± 0.10	0.169^b^
TCA (D)	0.90 ± 0.72	0.92 ± 0.69	0.02 ± 0.39	0.280^a^

Data are presented as mean ± standard deviation or as number (percent)

Statistically significant values (*p* < 0.05) are shown in asterisk (^*^)

^a^ Wilcoxon signed rank test

^b^ Paired t-test

*AL* axial length, *AK* anterior keratometry, *D* diopter, *PK* posterior keratometry, *TK* total keratometry, *ACA* anterior corneal astigmatism, *PCA* posterior corneal astigmatism, *TCA* total corneal astigmatism

**Table 3:** Longitudinal change in ocular biometry measured by IOL Master 700 in 147 eyes with against-the-rule astigmatism

Parameter	Initial measurement	Final measurement	Difference (Final-Initial)	*p*-value
Age (year)	70.56 ± 10.05	72.51 ± 10.09	1.95 ± 0.86	**< 0.001**^a*^
Sex (male/female)	64 (44%) / 83 (56%)		-	-
Laterality (right/left)	76 (52%) / 71 (48%)		-	-
AL (mm)	24.08 ± 1.48	24.11 ± 1.48	0.02 ± 0.04	**< 0.001**^b*^
Flat AK (D)	43.20 ± 1.48	43.19 ± 1.49	0.00 ± 0.33	0.655^a^
Steep AK (D)	44.15 ± 1.44	44.21 ± 1.44	0.06 ± 0.26	**0.005**^b*^
Mean AK (D)	43.67 ± 1.43	43.70 ± 1.43	0.03 ± 0.25	0.148^b^
Flat PK (D)	−5.79 ± 0.22	−5.79 ± 0.21	0.00 ± 0.08	0.685^b^
Steep PK (D)	−6.02 ± 0.24	−6.02 ± 0.24	0.00 ± 0.08	0.874^b^
Mean PK (D)	−5.91 ± 0.22	−5.91 ± 0.22	0.00 ± 0.06	0.875^b^
Flat TK (D)	43.02 ± 1.48	43.02 ± 1.49	0.00 ± 0.37	0.904^b^
Steep TK (D)	44.26 ± 1.45	44.32 ± 1.46	0.07 ± 0.29	**0.005**^a*^
Mean TK (D)	43.64 ± 1.44	43.67 ± 1.44	0.03 ± 0.27	0.124^b^
TK-AK (D)	−0.03 ± 0.11	−0.03 ± 0.10	0.00 ± 0.06	0.537^a^
ACA (D)	0.95 ± 0.50	1.02 ± 0.59	0.06 ± 0.35	**0.026**^b*^
PCA (D)	−0.23 ± 0.11	−0.23 ± 0.11	0.00 ± 0.10	0.651^b^
TCA (D)	1.24 ± 0.53	1.30 ± 0.65	0.06 ± 0.38	0.054^b^

Data are presented as mean ± standard deviation or as number (percent)

Statistically significant values (*p* < 0.05) are shown in asterisk (^*^)

^a^ Wilcoxon signed rank test

^b^ Paired t-test

*AL* axial length, *AK* anterior keratometry, *D* diopter, *PK* posterior keratometry, *TK* total keratometry, *ACA* anterior corneal astigmatism, *PCA* posterior corneal astigmatism, *TCA* total corneal astigmatism

**Fig. 1:**
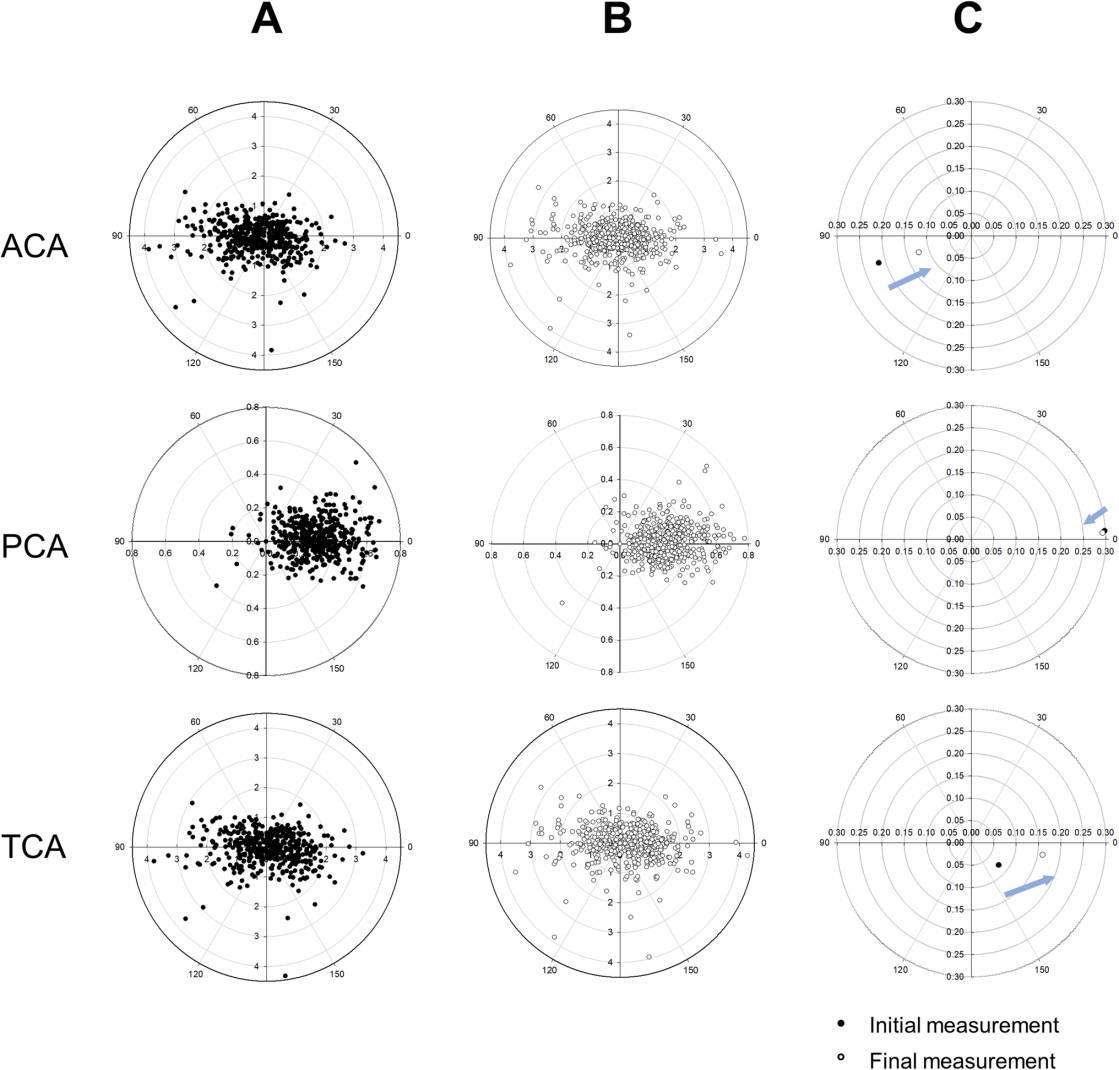
Double angle plots of anterior, posterior, and total astigmatism from (**A**) initial measurement, (**B)** final measurement, and (**C**) average of each. (**A**, **B**, **C**) Anterior corneal astigmatism showed with-the-rule (WTR) astigmatism at initial measurements, which remained WTR with decreased magnitude at final measurements. Posterior corneal astigmatism showed against-the-rule (ATR) astigmatism at initial measurements, which remained ATR with minimal change in the magnitude at final measurements. Total corneal astigmatism showed WTR astigmatism at initial measurement, which remained WTR with increased magnitude at final measurements

Double-angle plots of the ACA, PCA, and TCA from the initial and final measurements are illustrated in Fig. 1. Over the course of 23.4 months, the ACA, PCA, and TCA maintained WTR, ATR, and WTR astigmatism, respectively (Fig. 1C).

Of the 448 eyes, 119 eyes of the 119 patients underwent cataract surgery using Tecnis ZCB00, the most commonly used monofocal IOL at SNUH. The average time between consecutive biometric measurements was 23.6 month. The initial and final biometric measurements and their differences are presented in Supplementary Table 8. Ninety eyes (76%) were targeted for emmetropia, while 29 eyes (24%) were targeted for near vision (–1.5 D to − 3.0 D). A comparison of the ME from the initial and final measurements showed no significant differences in all formulas (Table 4). However, MAE of the final measurements were significantly smaller compared to the initial measurements in the BUII, EVO, Kane, and Pearl DGS formulas (difference of −0.05 D, −0.05 D, −0.06 D, and − 0.05 D, respectively). MAE from the two measurements revealed no differences in SRK/T, Cook K6, Hill-RBF and Hoffer QST formulas. Percentages of eyes with absolute predictive errors ≤ 0.25, ≤ 0.50, ≤ 0.75 and ≤ 1.00D were greater in the final measurements compared to the initial measurements in all formulas except SRK/T (Table 4). Specifically, in eyes with AL ≥ 25 mm, the MAE of the final measurements was significantly smaller than that of the initial measurements in the BUII, Cook K6, EVO, Hill-RBF, Hoffer QST, Kane, and Pearl DGS formulas, with differences of −0.11 D, −0.11 D, −0.10 D, −0.08 D, −0.10 D, −0.09 D and − 0.10 D, respectively. In contrast, no significant differences were observed between the two measurements in eyes with AL < 23 mm or AL between 23 and 25 mm (Table 5).

**Table 4:** Comparison of mean predictive errors and mean absolute predictive errors from initial and final measurements of IOL Master 700 taken at least one year apart in 119 eyes

Formula		ME ± SD	MAE ± SD	MedE	MedAE	%AE ≤ 0.25D	%AE ≤ 0.50D	%AE ≤ 0.75D	%AE ≤ 1.00D
SRK/T	Initial	−0.16 ± 0.48	0.38 ± 0.34	−0.11	0.28	49	69	86	93
	Final	−0.12 ± 0.46	0.35 ± 0.32	−0.09	0.27	45	81	87	94
	Difference	0.04 ± 0.22	−0.03 ± 0.19						
	*p*-value	0.137	0.215						
BUII	Initial	−0.10 ± 0.44	0.36 ± 0.28	−0.09	0.28	45	76	87	96
	Final	−0.07 ± 0.39	0.31 ± 0.25	−0.05	0.24	52	82	92	99
	Difference	0.03 ± 0.26	−0.05 ± 0.21						
	*p*-value	0.355	0.036^*^						
Cook K6	Initial	0.00 ± 0.41	0.32 ± 0.25	0.01	0.27	46	78	92	99
	Final	0.03 ± 0.36	0.28 ± 0.22	0.06	0.23	52	83	97	100
	Difference	0.03 ± 0.26	−0.04 ± 0.22						
	*p*-value	0.323	0.073						
EVO	Initial	−0.10 ± 0.42	0.33 ± 0.27	−0.10	0.26	50	76	90	98
	Final	−0.07 ± 0.36	0.28 ± 0.24	−0.03	0.22	55	82	95	100
	Difference	0.03 ± 0.25	−0.05 ± 0.21						
	*p*-value	0.270	0.036^*^						
Hill-RBF	Initial	−0.11 ± 0.43	0.34 ± 0.28	−0.10	0.27	49	73	88	97
	Final	−0.07 ± 0.39	0.31 ± 0.25	−0.06	0.23	54	80	93	98
	Difference	0.04 ± 0.25	−0.03 ± 0.21						
	*p*-value	0.224	0.189						
Hoffer QST	Initial	−0.11 ± 0.46	0.36 ± 0.31	−0.06	0.28	45	75	89	94
	Final	−0.07 ± 0.42	0.32 ± 0.27	−0.06	0.25	52	79	92	97
	Difference	0.04 ± 0.25	−0.04 ± 0.21						
	*p*-value	0.287	0.254						
Kane	Initial	−0.16 ± 0.42	0.34 ± 0.29	−0.12	0.27	48	77	89	96
	Final	−0.13 ± 0.36	0.28 ± 0.26	−0.06	0.21	59	81	92	99
	Difference	0.03 ± 0.25	−0.06 ± 0.21						
	*p*-value	0.370	0.008^*^						
Pearl DGS	Initial	−0.06 ± 0.43	0.34 ± 0.27	−0.03	0.24	52	77	89	98
	Final	−0.03 ± 0.36	0.29 ± 0.23	0.02	0.24	53	81	96	100
	Difference	0.03 ± 0.27	−0.05 ± 0.22						
	*p*-value	0.424	0.012^*^						

Data are presented as mean ± standard deviation

Statistically significant values (*p* < 0.05) are shown in asterisk (^*^)

Data were analyzed using Wilcoxon matched-pairs signed rank test

*ME* mean predictive error, *SD* standard deviation, *MAE* mean absolute predictive error, *MedE* median predictive error, *MedAE* median absolute predictive error, *AE* absolute predictive error, *BUII* Barrett Universal II

**Table 5:** Comparison of mean predictive errors and mean absolute predictive errors from initial and final measurements of IOL Master 700 taken at least one year apart in 119 eyes according to axial length

Formula	ME ± SD				MAE ± SD		
		< 23 mm (*N* = 19)	23–25 mm (*N* = 72)	≥ 25 mm (*N* = 28)	< 23 mm (*N* = 19)	23–25 mm (*N* = 72)	≥ 25 mm (*N* = 28)
SRK/T	Initial	−0.27 ± 0.46	−0.18 ± 0.49	−0.04 ± 0.48	0.41 ± 0.33	0.38 ± 0.35	0.37 ± 0.30
	Final	−0.23 ± 0.44	−0.14 ± 0.47	0.00 ± 0.43	0.38 ± 0.31	0.36 ± 0.33	0.30 ± 0.30
	Difference	0.04 ± 0.18	0.04 ± 0.22	0.04 ± 0.22	−0.03 ± 0.18	−0.02 ± 0.19	−0.07 ± 0.20
	*p*-value	0.339^a^	0.118^a^	0.736^b^	0.469^a^	0.751^b^	0.078^a^
BUII	Initial	−0.12 ± 0.44	−0.09 ± 0.42	−0.11 ± 0.51	0.35 ± 0.28	0.33 ± 0.28	0.43 ± 0.29
	Final	−0.11 ± 0.37	−0.05 ± 0.39	−0.08 ± 0.40	0.30 ± 0.25	0.31 ± 0.25	0.31 ± 0.26
	Difference	0.01 ± 0.22	0.04 ± 0.27	0.03 ± 0.26	−0.06 ± 0.19	−0.02 ± 0.21	−0.11 ± 0.23
	*p*-value	0.969^a^	0.216^a^	0.522^a^	0.197^a^	0.763^b^	**0.013**^a*^
Cook K6	Initial	0.09 ± 0.35	0.00 ± 0.39	−0.05 ± 0.46	0.29 ± 0.21	0.30 ± 0.25	0.37 ± 0.28
	Final	0.11 ± 0.30	0.03 ± 0.37	−0.01 ± 0.36	0.25 ± 0.20	0.30 ± 0.22	0.26 ± 0.24
	Difference	0.03 ± 0.19	0.03 ± 0.27	0.04 ± 0.29	−0.04 ± 0.15	−0.01 ± 0.22	−0.11 ± 0.25
	*p*-value	0.538^a^	0.307^a^	0.451^a^	0.247^a^	0.715^a^	**0.021**^a*^
EVO	Initial	−0.11 ± 0.37	−0.09 ± 0.42	−0.13 ± 0.46	0.29 ± 0.24	0.32 ± 0.27	0.38 ± 0.28
	Final	−0.08 ± 0.32	−0.06 ± 0.38	−0.09 ± 0.36	0.24 ± 0.22	0.30 ± 0.24	0.27 ± 0.25
	Difference	0.03 ± 0.19	0.03 ± 0.27	0.04 ± 0.26	−0.05 ± 0.18	−0.03 ± 0.20	−0.10 ± 0.22
	*p*-value	0.506^a^	0.306^a^	0.366^a^	0.212^a^	0.289^a^	**0.002**^b*^
Hill-RBF	Initial	−0.05 ± 0.39	−0.09 ± 0.42	−0.18 ± 0.48	0.31 ± 0.23	0.32 ± 0.29	0.41 ± 0.30
	Final	−0.03 ± 0.35	−0.05 ± 0.40	−0.15 ± 0.40	0.26 ± 0.23	0.31 ± 0.25	0.33 ± 0.26
	Difference	0.03 ± 0.19	0.04 ± 0.26	0.04 ± 0.27	−0.05 ± 0.17	−0.01 ± 0.21	−0.08 ± 0.24
	*p*-value	0.537^a^	0.191^a^	0.495^a^	0.232^a^	0.657^a^	**0.031**^b*^
Hoffer QST	Initial	−0.16 ± 0.42	−0.06 ± 0.45	−0.20 ± 0.50	0.34 ± 0.28	0.34 ± 0.31	0.41 ± 0.34
	Final	−0.13 ± 0.38	−0.02 ± 0.43	−0.18 ± 0.39 99	0.29 ± 0.26	0.33 ± 0.27	0.31 ± 0.30
	Difference	0.04 ± 0.20	0.04 ± 0.26	0.02 ± 0.26	−0.05 ± 0.19	0.00 ± 0.21	−0.10 ± 0.23
	*p*-value	0.449^a^	0.191^a^	0.653^a^	0.246^a^	0.605^b^	**0.022**^a*^
Kane	Initial	−0.13 ± 0.36	−0.14 ± 0.41	−0.22 ± 0.47	0.29 ± 0.25	0.33 ± 0.28	0.40 ± 0.33
	Final	−0.10 ± 0.31	−0.11 ± 0.37	−0.18 ± 0.38	0.23 ± 0.23	0.28 ± 0.26	0.31 ± 0.28
	Difference	0.03 ± 0.19	0.03 ± 0.26	0.04 ± 0.26	−0.05 ± 0.19	−0.05 ± 0.21	−0.09 ± 0.22
	*p*-value	0.535^a^	0.320^a^	0.395^a^	0.221^a^	0.064^a^	**0.011**^b*^
Pearl DGS	Initial	0.08 ± 0.38	−0.03 ± 0.41	−0.21 ± 0.48	0.31 ± 0.22	0.32 ± 0.25	0.41 ± 0.32
	Final	0.11 ± 0.33	−0.01 ± 0.36	−0.17 ± 0.37	0.26 ± 0.22	0.29 ± 0.21	0.30 ± 0.27
	Difference	0.03 ± 0.20	0.03 ± 0.29	0.04 ± 0.28	−0.05 ± 0.18	−0.03 ± 0.22	−0.10 ± 0.24
	*p*-value	0.592^a^	0.397^a^	0.416^a^	0.268^a^	0.212^a^	**0.003**^b*^

Data are presented as mean ± standard deviation

Statistically significant values (*p* < 0.05) are shown in asterisk (^*^)

^a^ Paired t-test

^b^ Wilcoxon signed rank test

*ME* mean predictive error, *SD* standard deviation, *MAE* mean absolute predictive error, *BUII* Barrett Universal II

## Discussion

We investigated changes in ocular biometry over time and assessed their impact on the accuracy of IOL power calculations. Ocular biometry measured at an average interval of 23.4 months showed that as age increased, AL increased and TK of flat axes decreased, resulting in an increase in the magnitude of TCA. Moreover, biometric measurements performed within one month prior to cataract surgery showed a significantly lower predictive error for postoperative refraction when calculated using the BUII, EVO, Kane, and Pearl DGS formulas compared to initial measurements. Specifically, eyes with longer AL (≥ 25 mm) showed significant difference between the two measurements, with the final biometry yielding significantly lower predictive errors in the BUII, Cook K6, EVO, Hill-RBF, Hoffer QST, Kane, and Pearl DGS formulas. This is the first study to investigate the longitudinal changes in ocular biometry measured by the IOLMaster 700 in an adult Korean population, as well as the first to compare postoperative refractive error with biometric measurements taken over one year apart. Our findings suggest that IOLMaster 700 measurements taken within one month before cataract surgery are more accurate than those taken more than those taken over a year prior when using the BUII, EVO, Kane, and Pearl DGS formulas. If a scheduled surgery is delayed for more than a year, it is advisable to reassess ocular biometry using the IOLMaster 700 to enhance the accuracy of postoperative refraction with the aforementioned formulas.

A previous report from our group involving 5,273 eyes of an adult Korean population with cataracts showed that age was negatively correlated with AL, ACD, PK, ACA, PCA, and TCA and positively correlated with LT, AK, TK, angle alpha, and angle kappa [17]. This is in contrast with our results, which showed that AL and TCA significantly increased with age. The former was a cross-sectional study limited by the fact that changes in ocular biometric parameters in the population may not be reproducible at the individual level. This is likely due to a rise in myopia among younger individuals, influenced by increased engagement in short-distance work and decreased outdoor activities, driven by rapid industrialization and urbanization in Korea [17]. As our longitudinal study focused on individual changes over time, this confounding effect was not a factor in the present analysis, allowing for a more accurate depiction of AL change over time.

Several longitudinal studies on changes in ocular biometry have been published. A study in United States by Kuriakose et al. [15] involved 402 eyes of 201 patients over 35 years old. They found no significant changes in AL, keratometry (K), ACD, or LT during the average period of 21.5 months. Although it did not reach statistical significance, mean change in AL was + 0.04 mm, which is consistent with our results. Kuriakose et al. noted that the recommended IOL power was similar between the two biometric measurements when using BUII or SRK/T formulas, though specific data were not provided. Another longitudinal cohort study in United Kingdom revealed that both flat K and steep K significantly increased by + 0.06 D and + 0.33 D, respectively, while AL remained unchanged in 70 eyes measured more than 24 months apart [14]. In contrast, our results showed a significant decrease in flat TK by − 0.02 D with no definite change in steep TK and a significant increase in AL. The difference between the two studies may be due to the smaller cohort and the switch in the biometry measurement device during the study period, transitioning from the IOLMaster in the former period to the IOLMaster 500 in the latter in the study by Rufai et al. Additionally, the considerably older and racially diverse cohort in the United Kingdom may have played a role. In a prospective cohort study of 1300 Chinese adults aged 35 years or older, Han et al. [13] investigated longitudinal changes in refraction and biometry over a 6 year period. The study demonstrated that AL, K, and LT increased, ACD decreased, and the axis of astigmatism changed from WTR to ATR in 16.4% of the participants and to oblique in 0.9%. Although the observed increase in AL was consistent with our findings, in contrast to the study by Han et al., our cohort showed no notable changes in K, ACD, or LT, and the average ACA vector remained at WTR. These differences may be attributed to the younger age group, longer follow-up period, or the inconsistent use of biometric measurement devices in the Chinese study.

New-generation formulas, including Kane, BUII, Cook K6, EVO, Hill-RBF, Hoffer QST, and Pearl DGS, have demonstrated superior accuracy compared to previous generations [18–23]. Consistent with these findings, our study showed that the new-generation formulas exhibited a lower MAE than SRK/T. In addition, higher percentages of eyes with absolute predictive error ≤ 0.25 D, ≤ 0.50 D, ≤ 0.75 D, and ≤ 1.00 D were observed with new-generation formulas compared to SRK/T. Moreover, biometric measurements performed within one month prior to cataract surgery showed a lower predictive error for postoperative refraction when calculated using the BUII, EVO, Kane, and Pearl DGS formulas compared to initial measurements. This result may be attributed to the fact that new generation formulas are more sensitive to longitudinal changes in ocular biometry, as they incorporate more biometric parameters.

An interesting and important finding from our study is that the final biometric measurements significantly improved postoperative refractive accuracy compared to the initial measurements in myopic eyes (AL ≥ 25 mm), whereas hyperopic eyes (AL < 23 mm) and eyes with normal AL (AL between 23 and 25 mm) showed no significant differences. This improvement may be attributed to the continued elongation of AL in adults with high myopia, as reported in previous studies [24, 25]. With reductions in MAE of 0.11 D (BUII), 0.11 D (Cook K6), 0.10 D (EVO), 0.08 D (Hill-RBF), 0.10 D (Hoffer QST), 0.09 D (Kane) and 0.10 D (Pearl DGS), the magnitude of these differences underscores the necessity of repeating ocular biometry before surgery, particularly in eyes with longer AL.

The limitations of our study include the relatively small sample size, with 448 eyes whose biometric measurements were separated by an interval of one year or more. Another limitation is that our predictive refraction and predictive error analyses were restricted to patients who received the ZCB00 IOL. In the future, exploring a larger cohort with extended time intervals between biometric measurements and evaluating how repeated biometry for toric or multifocal IOLs affects predictive refraction and predictive error would be of great interest.

In this cohort of cataract patients who were observed for at least one year and for an average of 23.4 months, changes in AL and corneal curvature were observed with increasing age. Biometric measurements performed within one month before cataract surgery showed significantly improved accuracy when calculated using the BUII, EVO, Kane, and Pearl DGS formulas compared to measurements taken over one year ago. Therefore, it is recommended to repeat IOLMaster 700 biometry before surgery if the previous measurements were obtained more than a year ago when using these IOL power formulas.

## Supplementary Information

Below is the link to the electronic supplementary material.

ESM 1 (DOCX 109 KB)

## Data Availability

The datasets generated during and/or analyzed during the current study are available from the corresponding author on reasonable request.

## References

[1] Kauh CY, Blachley TS, Lichter PR et al (2016) Geographic Variation in the rate and timing of cataract surgery among US communities. JAMA Ophthalmol 134(3):267–276 26720865 10.1001/jamaophthalmol.2015.5322 PMC5767078

[2] Chen X, Xu J, Chen X, Yao K (2021) Cataract: advances in surgery and whether surgery remains the only treatment in future. Adv Ophthalmol Pract Res 1(1):100008 37846393 10.1016/j.aopr.2021.100008 PMC10577864

[3] Lee AC, Qazi MA, Pepose JS (2008) Biometry and intraocular lens power calculation. Curr Opin Ophthalmol 19(1):13–17 18090891 10.1097/ICU.0b013e3282f1c5ad

[4] Sahin A, Hamrah P (2012) Clinically relevant biometry. Curr Opin Ophthalmol 23(1):47–53 22081032 10.1097/ICU.0b013e32834cd63e PMC3299090

[5] Montés-Micó R, Pastor-Pascual F, Ruiz-Mesa R, Tañá-Rivero P (2021) Ocular biometry with swept-source optical coherence tomography. J Cataract Refract Surg 47(6):802–814 33315731 10.1097/j.jcrs.0000000000000551

[6] Hirnschall N, Varsits R, Doeller B, Findl O (2018) Enhanced penetration for axial length measurement of eyes with dense cataracts using swept source Optical Coherence Tomography: a consecutive observational study. Ophthalmol Ther 7(1):119–124 29498015 10.1007/s40123-018-0122-1 PMC5997603

[7] Fabian E, Wehner W (2019) Prediction accuracy of total Keratometry compared to Standard Keratometry using different intraocular Lens Power Formulas. J Refract Surg 35(6):362–368 31185101 10.3928/1081597X-20190422-02

[8] Ferrer-Blasco T, Domínguez-Vicent A, Esteve-Taboada JJ et al (2017) Evaluation of the repeatability of a swept-source ocular biometer for measuring ocular biometric parameters. Graefes Arch Clin Exp Ophthalmol 255(2):343–349 27900479 10.1007/s00417-016-3555-z

[9] Savini G, Taroni L, Schiano-Lomoriello D, Hoffer KJ (2021) Repeatability of total Keratometry and standard Keratometry by the IOLMaster 700 and comparison to total corneal astigmatism by Scheimpflug imaging. Eye (Lond) 35(1):307–315 33139878 10.1038/s41433-020-01245-8 PMC7852681

[10] Borgia A, Raimondi R, Sorrentino T et al (2022) Swept-source Optical Coherence Tomography-based Biometry: a comprehensive overview. Photonics 9(12):951

[11] Jung S, Chin HS, Kim NR et al (2017) *Comparison of Repeatability and Agreement between Swept-Source Optical Biometry and Dual-Scheimpflug Topography.* J Ophthalmol, 2017: p. 1516395 10.1155/2017/1516395 PMC5742459 29375908

[12] Hashemi H, Khabazkhoob M, Iribarren R et al (2016) Five-year change in refraction and its ocular components in the 40- to 64-year-old population of the Shahroud eye cohort study. Clin Exp Ophthalmol 44(8):669–677 27059537 10.1111/ceo.12753

[13] Han X, Guo X, Lee PY et al (2017) Six-year changes in refraction and related ocular biometric factors in an adult Chinese population. PLoS ONE 12(8):e0183364 28854269 10.1371/journal.pone.0183364 PMC5576680

[14] Rufai SR, Moghaddam YB, Menon VJ (2020) Do biometric readings change significantly over Time in phakic eyes? A cohort study. Semin Ophthalmol 35(7–8):343–347 33370159 10.1080/08820538.2020.1862875

[15] Kuriakose RK, Gulati R, Bains H et al (2023) Change in major ocular biometry parameters axial length and keratometry in adults over time. J Cataract Refract Surg 49(5):474–478 36700942 10.1097/j.jcrs.0000000000001144

[16] Armstrong RA (2013) Statistical guidelines for the analysis of data obtained from one or both eyes. Ophthalmic Physiol Opt 33(1):7–14 23252852 10.1111/opo.12009

[17] Kim S, Oh R, Kim MK, Yoon CH (2023) SS-OCT-based ocular biometry in an adult Korean population with cataract. J Cataract Refract Surg 49(5):453–459 36700930 10.1097/j.jcrs.0000000000001135

[18] Yoon JH, Whang WJ (2023) Comparison of accuracy of six modern intraocular Lens Power calculation formulas. Korean J Ophthalmol 37(5):380–386 37562439 10.3341/kjo.2023.0034 PMC10587458

[19] Nemeth G, Kemeny-Beke A, Modis L Jr. (2022) Comparison of accuracy of different intraocular lens power calculation methods using artificial intelligence. Eur J Ophthalmol 32(1):235–241 33594897 10.1177/1120672121994720

[20] Shammas HJ, Taroni L, Pellegrini M et al (2022) Accuracy of newer intraocular lens power formulas in short and long eyes using sum-of-segments biometry. J Cataract Refract Surg 48(10):1113–1120 35473887 10.1097/j.jcrs.0000000000000958 PMC9514730

[21] Hipólito-Fernandes D, Luís ME, Serras-Pereira R et al (2022) Anterior chamber depth, lens thickness and intraocular lens calculation formula accuracy: nine formulas comparison. Br J Ophthalmol 106(3):349–355 33229347 10.1136/bjophthalmol-2020-317822

[22] Voytsekhivskyy OV, Hoffer KJ, Tutchenko L et al (2023) Accuracy of 24 IOL Power Calculation Methods. J Refract Surg 39(4):249–256 37040214 10.3928/1081597X-20230131-01

[23] Taroni L, Hoffer KJ, Pellegrini M et al (2023) Comparison of the new Hoffer QST with 4 modern accurate formulas. J Cataract Refract Surg 49(4):378–384 36729423 10.1097/j.jcrs.0000000000001126

[24] Du R, Xie S, Igarashi-Yokoi T et al (2021) Continued increase of axial length and its risk factors in adults with high myopia. JAMA Ophthalmol 139(10):1096–1103 34436537 10.1001/jamaophthalmol.2021.3303 PMC8391777

[25] Lee MW, Lee SE, Lim HB, Kim JY (2020) Longitudinal changes in axial length in high myopia: a 4-year prospective study. Br J Ophthalmol 104(5):600–603 31409648 10.1136/bjophthalmol-2019-314619

