# Supplementary material for: Longitudinal changes in ocular biometry and their effect on intraocular lens power calculation accuracy in cataract patients
Source: Graefes Arch Clin Exp Ophthalmol. 2025 Feb 28;263(7):1915–24. doi: 10.1007/s00417-025-06775-z (PMC12373673; doi:10.1007/s00417-025-06775-z)
Supplement: Supplementary file 1 — (DOCX 109 KB) [file 417_2025_6775_MOESM1_ESM.docx]

**Supplementary Table 1.** Longitudinal change in ocular biometry measured by IOL Master 700 according to gender.

| Parameter | **Male**  (N = 161) | | | |  | **Female**  (N = 287) | | | |
| --- | --- | --- | --- | --- | --- | --- | --- | --- | --- |
|  | Initial  measurement | Final  measurement | Difference  (Final- Initial) | *p*-value |  | Initial  measurement | Final  measurement | Difference  (Final- Initial) | *p*-value |
| Age (year) | 63.88 ± 15.18 | 65.77 ± 15.25 | 1.89 ± 0.67 | **<0.001^a*^** |  | 64.72 ± 12.07 | 66.70 ± 12.02 | 1.98 ± 0.82 | **<0.001^a*^** |
| Sex (male/female) | 68 (42%) / 93 (58%) | |  |  |  | 138 (48%) / 149 (52%) | |  |  |
| AL (mm) | 25.13± 2.07 | 25.17 ± 2.09 | 0.03 ± 0.07 | **<0.001^a*^** |  | 24.27 ± 1.97 | 24.31 ± 2.00 | 0.04 ± 0.11 | **<0.001^a*^** |
| Flat AK (D) | 42.89 ± 1.52 | 42.86 ± 1.55 | -0.03 ± 0.30 | 0.074^a^ |  | 43.81 ± 1.46 | 43.81 ± 1.47 | 0.00 ± 0.27 | 0.577^a^ |
| Steep AK (D) | 43.87 ± 1.54 | 43.86 ± 1.54 | -0.01 ± 0.29 | 0.805^a^ |  | 44.76 ± 1.46 | 44.77 ± 1.46 | 0.01 ± 0.31 | 0.753^a^ |
| Mean AK (D) | 43.38 ± 1.49 | 43.36 ± 1.50 | -0.02 ± 0.23 | 0.292^b^ |  | 44.29 ± 1.43 | 44.29 ± 1.43 | 0.00 ± 0.24 | 0.806^b^ |
| Flat PK (D) | -5.69 ± 0.25 | -5.68 ± 0.24 | 0.01 ± 0.07 | 0.294^b^ |  | -5.81 ± 0.22 | -5.82 ± 0.22 | 0.00 ± 0.07 | 0.472^b^ |
| Steep PK (D) | -6.00 ± 0.26 | -6.00 ± 0.26 | 0.01 ± 0.07 | 0.266^b^ |  | -6.14 ± 0.25 | -6.14 ± 0.25 | 0.00 ± 0.09 | 0.961^a^ |
| Mean PK (D) | -5.84 ± 0.24 | -5.84 ± 0.24 | 0.01 ± 0.06 | 0.159^b^ |  | -5.98 ± 0.23 | -5.98 ± 0.23 | 0.00 ± 0.06 | 0.943^b^ |
| Flat TK (D) | 42.85 ± 1.52 | 42.80 ± 1.54 | -0.05 ± 0.34 | 0.085^b^ |  | 43.78 ± 1.47 | 43.78 ± 1.47 | -0.01 ± 0.32 | 0.344^a^ |
| Steep TK (D) | 43.89 ± 1.54 | 43.90 ± 1.55 | 0.02 ± 0.31 | 0.295^a^ |  | 44.75 ± 1.45 | 44.77 ± 1.46 | 0.02 ± 0.32 | 0.404^b^ |
| Mean TK (D) | 43.37 ± 1.49 | 43.35 ± 1.50 | -0.02 ± 0.26 | 0.579^a^ |  | 44.27 ± 1.43 | 44.27 ± 1.43 | 0.00 ± 0.26 | 0.780^b^ |
| TK-AK (D) | -0.01 ± 0.12 | -0.01 ± 0.12 | 0.00 ± 0.06 | 0.418^b^ |  | -0.02 ± 0.10 | -0.02 ± 0.11 | 0.00 ± 0.06 | 0.819^b^ |
| ACA (D) | 0.98 ± 0.68 | 1.00 ± 0.73 | 0.02 ± 0.36 | 0.486^b^ |  | 0.95 ± 0.64 | 0.96 ± 0.64 | 0.01 ± 0.35 | 0.529^b^ |
| PCA (D) | -0.32 ± 0.15 | -0.32 ± 0.15 | 0.00 ± 0.09 | 0.562^a^ |  | -0.33 ± 0.14 | -0.32 ± 0.14 | 0.01 ± 0.10 | 0.260^b^ |
| TCA (D) | 1.04 ± 0.70 | 1.10 ± 0.76 | 0.06 ± 0.40 | **0.016^a*^** |  | 0.96 ± 0.64 | 0.99 ± 0.64 | 0.02 ± 0.38 | 0.250^a^ |
| CCT (mm) | 0.55 ± 0.04 | 0.55 ± 0.04 | 0.00 ± 0.01 | 0.066^b^ |  | 0.54 ± 0.03 | 0.54 ± 0.03 | 0.00 ± 0.01 | 0.684^b^ |
| ACD (mm) | 3.26 ± 0.40 | 3.27 ± 0.43 | 0.00 ± 0.10 | 0.582^a^ |  | 3.06 ± 0.42 | 3.06 ± 0.42 | 0.00 ± 0.09 | 0.999^a^ |
| LT (mm) | 4.44 ± 0.46 | 4.43 ± 0.49 | -0.01 ± 0.17 | 0.158^a^ |  | 4.47 ± 0.49 | 4.45 ± 0.51 | -0.02 ± 0.42 | 0.249^a^ |
| WTW(mm) | 11.92 ± 0.47 | 11.92 ± 0.50 | 0.00 ± 0.35 | 0.900^a^ |  | 11.71 ± 0.40 | 11.72 ± 0.40 | 0.01 ± 0.29 | 0.220^a^ |
| Angle alpha (mm) | 0.42 ± 0.21 | 0.43 ± 0.26 | 0.01 ± 0.27 | 0.763^a^ |  | 0.44 ± 0.22 | 0.45 ± 0.24 | 0.01 ± 0.22 | 0.569^a^ |
| Angle kappa (mm) | 0.26 ± 0.14 | 0.26 ± 0.14 | 0.00 ± 0.14 | 0.390^a^ |  | 0.25 ± 0.15 | 0.27 ± 0.15 | 0.01 ± 0.13 | 0.258^a^ |

Data are presented as mean ± standard deviation or as number (percent)

Statistically significant values (*p* < 0.05) are shown in asterisk (^*^)

^a^ Wilcoxon signed rank test

^b^ Paired t-test

AL, axial length; AK, anterior keratometry; D, diopter; PK, posterior keratometry; TK, total keratometry; ACA, anterior corneal astigmatism; PCA, posterior corneal astigmatism; TCA, total corneal astigmatism; CCT, central corneal thickness; ACD, anterior chamber depth; LT, lens thickness; WTW, white-to-white distance

**Supplementary Table 2.** Longitudinal change in ocular biometry measured by IOL Master 700 according to age.

| Parameter | **Age < 50 years**  (N = 59) | | | |  | **Age ≥ 50 years**  (N = 389) | | | |
| --- | --- | --- | --- | --- | --- | --- | --- | --- | --- |
|  | Initial  measurement | Final  measurement | Difference  (Final- Initial) | *p*-value |  | Initial  measurement | Final  measurement | Difference  (Final- Initial) | *p*-value |
| Age (year) | 38.62 ± 9.17 | 40.53 ± 9.20 | 1.91 ± 0.80 | **<0.001^a*^** |  | 68.33 ± 8.57 | 70.28 ± 8.54 | 1.95 ± 0.77 | **<0.001^a*^** |
| Sex (male/female) | 29 (49%) / 30 (51%) | |  |  |  | 132 (34%) / 257(66%) | |  |  |
| Laterality (right/left) | 30 (51%) / 29 (49%) | |  |  |  | 176 (45%) / 213 (55%) | |  |  |
| AL (mm) | 26.87 ± 2.49 | 26.93 ± 2.55 | 0.06 ± 0.11 | **<0.001^a*^** |  | 24.23 ± 1.72 | 24.26 ± 1.75 | 0.03 ± 0.09 | **<0.001^a*^** |
| Flat AK (D) | 42.38 ± 1.65 | 42.38 ± 1.66 | 0.00 ± 0.27 | 0.939^b^ |  | 43.65 ± 1.47 | 43.63 ± 1.48 | -0.01 ± 0.29 | 0.114^a^ |
| Steep AK (D) | 43.67 ± 1.73 | 43.70 ± 1.77 | 0.03 ± 0.33 | 0.925^a^ |  | 44.56 ± 1.49 | 44.56 ± 1.49 | 0.00 ± 0.30 | 0.918^a^ |
| Mean AK (D) | 43.03 ± 1.65 | 43.04 ± 1.67 | 0.01 ± 0.25 | 0.802^a^ |  | 44.10 ± 1.44 | 44.09 ± 1.45 | -0.01 ± 0.23 | 0.569^a^ |
| Flat PK (D) | -5.59 ± 0.25 | -5.61 ± 0.25 | -0.01 ± 0.08 | 0.184^b^ |  | -5.80 ± 0.23 | -5.79 ± 0.23 | 0.00 ± 0.07 | 0.520^b^ |
| Steep PK (D) | -5.97 ± 0.29 | -5.97 ± 0.28 | -0.01 ± 0.06 | 0.437^b^ |  | -6.11 ± 0.25 | -6.10 ± 0.26 | 0.01 ± 0.09 | 0.288^a^ |
| Mean PK (D) | -5.78 ± 0.26 | -5.79 ± 0.25 | -0.01 ± 0.06 | 0.186^b^ |  | -5.95 ± 0.23 | -5.95 ± 0.23 | 0.00 ± 0.06 | 0.163^b^ |
| Flat TK (D) | 42.45 ± 1.65 | 42.44 ± 1.67 | -0.01 ± 0.30 | 0.791^b^ |  | 43.60 ± 1.48 | 43.58 ± 1.50 | -0.02 ± 0.33 | **0.031^a*^** |
| Steep TK (D) | 43.62 ± 1.71 | 43.64 ± 1.77 | 0.02 ± 0.36 | 0.843^a^ |  | 44.56 ± 1.48 | 44.58 ± 1.48 | 0.02 ± 0.31 | 0.256^a^ |
| Mean TK (D) | 43.04 ± 1.64 | 43.04 ± 1.68 | 0.00 ± 0.28 | 0.587^a^ |  | 44.08 ± 1.44 | 44.08 ± 1.45 | 0.00 ± 0.26 | 0.769^b^ |
| TK-AK (D) | 0.01 ± 0.12 | 0.00 ± 0.12 | -0.01 ± 0.06 | 0.318^b^ |  | -0.02 ± 0.11 | -0.02 ± 0.11 | 0.00 ± 0.06 | 0.467^a^ |
| ACA (D) | 1.29 ± 0.74 | 1.32 ± 0.77 | 0.03 ± 0.35 | 0.517^b^ |  | 0.91 ± 0.63 | 0.92 ± 0.64 | 0.01 ± 0.35 | 0.457^b^ |
| PCA (D) | -0.37 ± 0.13 | -0.37 ± 0.15 | 0.01 ± 0.08 | 0.531^a^ |  | -0.31 ± 0.14 | -0.31 ± 0.14 | 0.00 ± 0.10 | 0.452^b^ |
| TCA (D) | 1.17 ± 0.69 | 1.20 ± 0.73 | 0.03 ± 0.36 | 0.902^a^ |  | 0.96 ± 0.65 | 1.00 ± 0.68 | 0.04 ± 0.39 | **0.018^a*^** |
| CCT (mm) | 0.55 ± 0.03 | 0.55 ± 0.04 | 0.00 ± 0.01 | 0.167^b^ |  | 0.54 ± 0.03 | 0.54 ± 0.03 | 0.00 ± 0.01 | 0.325^b^ |
| ACD (mm) | 3.43 ± 0.41 | 3.38 ± 0.44 | -0.05 ± 0.13 | **0.007^b*^** |  | 3.09 ± 0.41 | 3.10 ± 0.42 | 0.01 ± 0.09 | 0.095^a^ |
| LT (mm) | 4.00 ± 0.40 | 4.00 ± 0.57 | 0.01 ± 0.45 | **<0.001^a*^** |  | 4.53 ± 0.45 | 4.51 ± 0.45 | -0.02 ± 0.34 | 0.626^a^ |
| WTW(mm) | 12.04 ± 0.46 | 11.95 ± 0.60 | -0.09 ± 0.46 | 0.429^a^ |  | 11.74 ± 0.42 | 11.77 ± 0.42 | 0.02 ± 0.28 | 0.195^a^ |
| Angle alpha (mm) | 0.36 ± 0.22 | 0.37 ± 0.27 | 0.01 ± 0.23 | 0.878^a^ |  | 0.45 ± 0.21 | 0.46 ± 0.25 | 0.01 ± 0.24 | 0.678^a^ |
| Angle kappa (mm) | 0.29 ± 0.15 | 0.28 ± 0.15 | -0.01 ± 0.15 | 0.756^a^ |  | 0.25 ± 0.14 | 0.26 ± 0.15 | 0.01 ± 0.13 | 0.569^a^ |

Data are presented as mean ± standard deviation or as number (percent)

Statistically significant values (*p* < 0.05) are shown in asterisk (^*^)

^a^ Wilcoxon signed rank test

^b^ Paired t-test

AL, axial length; AK, anterior keratometry; D, diopter; PK, posterior keratometry; TK, total keratometry; ACA, anterior corneal astigmatism; PCA, posterior corneal astigmatism; TCA, total corneal astigmatism; CCT, central corneal thickness; ACD, anterior chamber depth; LT, lens thickness; WTW, white-to-white distance

**Supplementary Table 3.** Longitudinal change in ocular biometry measured by IOL Master 700 according to presence of underlying hypertension.

| Parameter | **Hypertension (+)**  (N = 198) | | | |  | **Hypertension (-)**  (N = 250) | | | |
| --- | --- | --- | --- | --- | --- | --- | --- | --- | --- |
|  | Initial  measurement | Final  measurement | Difference  (Final- Initial) | *p*-value |  | Initial  measurement | Final  measurement | Difference  (Final- Initial) | *p*-value |
| Age (year) | 69.44 ± 9.51 | 71.42 ± 9.48 | 1.98 ± 0.77 | **<0.001^a*^** |  | 60.44 ± 14.43 | 42.36 ± 14.43 | 1.92 ± 0.78 | **<0.001^a*^** |
| Sex (male/female) | 75 (38%) / 123 (62%) | |  |  |  | 86 (34%) / 164 (66%) | |  |  |
| Laterality (right/left) | 93 (47%) / 105 (53%) | |  |  |  | 113 (45%) / 137 (55%) | |  |  |
| AL (mm) | 24.00 ± 1.43 | 24.03 ± 1.46 | 0.04 ± 0.12 | **<0.001^a*^** |  | 25.04 ± 2.30 | 25.08 ± 2.33 | 0.04 ± 0.07 | **<0.001^a*^** |
| Flat AK (D) | 43.69 ± 1.41 | 43.67 ± 1.42 | -0.02 ± 0.30 | 0.423^b^ |  | 43.32 ± 1.64 | 43.31 ± 1.65 | -0.01 ± 0.27 | 0.235^a^ |
| Steep AK (D) | 44.61 ± 1.39 | 44.63 ± 1.38 | 0.02 ± 0.30 | 0.473^b^ |  | 44.30 ± 1.66 | 44.30 ± 1.67 | -0.01 ± 0.30 | 0.778^a^ |
| Mean AK (D) | 44.15 ± 1.37 | 44.15 ± 1.36 | 0.00 ± 0.23 | 0.960^b^ |  | 43.81 ± 1.61 | 43.80 ± 1.63 | -0.01 ± 0.24 | 0.597^b^ |
| Flat PK (D) | -5.80 ± 0.22 | -5.80 ± 0.22 | 0.00 ± 0.07 | 0.756^b^ |  | -5.74 ± 0.25 | -5.74 ± 0.24 | 0.00 ± 0.08 | 0.858^b^ |
| Steep PK (D) | -6.11 ± 0.24 | -6.10 ± 0.25 | 0.01 ± 0.09 | 0.224^a^ |  | -6.07 ± 0.28 | -6.07 ± 0.28 | 0.00 ± 0.08 | 0.722^b^ |
| Mean PK (D) | -5.96 ± 0.22 | -5.95 ± 0.23 | 0.00 ± 0.06 | 0.280^b^ |  | -5.91 ± 0.25 | -5.91 ± 0.25 | 0.00 ± 0.06 | 0.904^b^ |
| Flat TK (D) | 43.64 ± 1.44 | 43.61 ± 1.44 | -0.03 ± 0.34 | 0.203^a^ |  | 43.30 ± 1.63 | 43.28 ± 1.65 | -0.02 ± 0.32 | 0.069^a^ |
| Steep TK (D) | 44.63 ± 1.38 | 44.66 ± 1.37 | 0.03 ± 0.32 | 0.123^b^ |  | 44.29 ± 1.65 | 44.29 ± 1.67 | 0.00 ± 0.32 | 0.828^a^ |
| Mean TK (D) | 44.13 ± 1.37 | 44.14 ± 1.36 | 0.00 ± 0.25 | 0.810^b^ |  | 43.80 ± 1.60 | 43.79 ± 1.63 | -0.01 ± 0.26 | 0.611^b^ |
| TK-AK (D) | -0.02 ± 0.11 | -0.01 ± 0.12 | 0.01 ± 0.06 | 0.438^a^ |  | -0.02 ± 0.11 | -0.02 ± 0.11 | 0.00 ± 0.06 | 0.874^b^ |
| ACA (D) | 0.93 ± 0.61 | 0.96 ± 0.66 | 0.03 ± 0.38 | 0.229^b^ |  | 0.99 ± 0.68 | 0.99 ± 0.67 | 0.00 ± 0.33 | 0.921^b^ |
| PCA (D) | -0.31 ± 0.14 | -0.30 ± 0.14 | 0.01 ± 0.10 | 0.344^b^ |  | -0.33 ± 0.14 | -0.33 ± 0.15 | 0.00 ± 0.10 | 0.679^b^ |
| TCA (D) | 0.99 ± 0.64 | 1.05 ± 0.71 | 0.06 ± 0.41 | **0.039^b*^** |  | 0.99 ± 0.68 | 1.01 ± 0.66 | 0.02 ± 0.36 | 0.311^a^ |
| CCT (mm) | 0.55 ± 0.03 | 0.55 ± 0.04 | 0.00 ± 0.01 | **0.002^a*^** |  | 0.54 ± 0.03 | 0.54 ± 0.03 | 0.00 ± 0.01 | 0.655^b^ |
| ACD (mm) | 3.06 ± 0.41 | 3.07 ± 0.43 | 0.01 ± 0.09 | **0.032^a*^** |  | 3.19 ± 0.43 | 3.19 ± 0.43 | -0.01 ± 0.10 | 0.146^a^ |
| LT (mm) | 4.58 ± 0.53 | 4.59 ± 0.43 | 0.01 ± 0.35 | 0.858^a^ |  | 4.37 ± 0.42 | 4.33 ± 0.53 | -0.03 ± 0.35 | **0.032^a*^** |
| WTW(mm) | 11.74 ± 0.41 | 11.74 ± 0.46 | 0.00 ± 0.36 | 0.593^a^ |  | 11.82 ± 0.45 | 11.83 ± 0.44 | 0.01 ± 0.27 | 0.428^a^ |
| Angle alpha (mm) | 0.46 ± 0.22 | 0.48 ± 0.27 | 0.02 ± 0.27 | 0.930^a^ |  | 0.42 ± 0.21 | 0.42 ± 0.23 | 0.00 ± 0.21 | 0.725^a^ |
| Angle kappa (mm) | 0.24 ± 0.13 | 0.25 ± 0.14 | 0.01 ± 0.12 | 0.784^a^ |  | 0.27 ± 0.16 | 0.27 ± 0.15 | 0.00 ± 0.15 | 0.768^a^ |

Data are presented as mean ± standard deviation or as number (percent)

Statistically significant values (*p* < 0.05) are shown in asterisk (^*^)

^a^ Wilcoxon signed rank test

^b^ Paired t-test

AL, axial length; AK, anterior keratometry; D, diopter; PK, posterior keratometry; TK, total keratometry; ACA, anterior corneal astigmatism; PCA, posterior corneal astigmatism; TCA, total corneal astigmatism; CCT, central corneal thickness; ACD, anterior chamber depth; LT, lens thickness; WTW, white-to-white distance

**Supplementary Table 4.** Longitudinal change in ocular biometry measured by IOL Master 700 according to presence of underlying diabetes.

| Parameter | **Diabetes (+)**  (N = 114) | | | |  | **Diabetes (-)**  (N = 334) | | | |
| --- | --- | --- | --- | --- | --- | --- | --- | --- | --- |
|  | Initial  measurement | Final  measurement | Difference  (Final- Initial) | *p*-value |  | Initial  measurement | Final  measurement | Difference  (Final- Initial) | *p*-value |
| Age (year) | 69.35 ± 9.11 | 71.30 ± 9.17 | 1.95 ± 0.74 | **<0.001^a*^** |  | 62.73 ± 14.02 | 64.68 ± 14.01 | 1.95 ± 0.78 | **<0.001^a*^** |
| Sex (male/female) | 45 (39%) / 69 (61%) | |  |  |  | 116 (35%) / 218 (65%) | |  |  |
| Laterality (right/left) | 49 (43%) / 65 (57%) | |  |  |  | 157 (47%) / 177 (53%) | |  |  |
| AL (mm) | 23.99 ± 1.39 | 24.01 ± 1.39 | 0.03 ± 0.05 | **<0.001^a*^** |  | 24.78 ± 2.19 | 24.82 ± 2.23 | 0.04 ± 0.11 | **<0.001^a*^** |
| Flat AK (D) | 43.70 ± 1.40 | 43.70 ± 1.41 | 0.00 ± 0.30 | 0.765^a^ |  | 43.41 ± 1.59 | 43.39 ± 1.61 | -0.02 ± 0.28 | 0.110^a^ |
| Steep AK (D) | 44.71 ± 1.41 | 44.74 ± 1.39 | 0.03 ± 0.30 | 0.320^b^ |  | 44.35 ± 1.59 | 44.34 ± 1.59 | -0.01 ± 0.30 | 0.816^a^ |
| Mean AK (D) | 44.20 ± 1.36 | 44.22 ± 1.35 | 0.01 ± 0.24 | 0.535^b^ |  | 43.88 ± 1.56 | 43.87 ± 1.57 | -0.01 ± 0.23 | 0.322^a^ |
| Flat PK (D) | -5.82 ± 0.21 | -5.81 ± 0.20 | 0.01 ± 0.07 | 0.354^b^ |  | -5.75 ± 0.25 | -5.75 ± 0.25 | 0.00 ± 0.07 | 0.650^b^ |
| Steep PK (D) | -6.14 ± 0.25 | -6.13 ± 0.26 | 0.01 ± 0.08 | 0.708^a^ |  | -6.07 ± 0.26 | -6.07 ± 0.26 | 0.00 ± 0.08 | 0.542^a^ |
| Mean PK (D) | -5.98 ± 0.22 | -5.97 ± 0.22 | 0.01 ± 0.06 | 0.428^a^ |  | -5.91 ± 0.25 | -5.91 ± 0.25 | 0.00 ± 0.06 | 0.864^b^ |
| Flat TK (D) | 43.61 ± 1.44 | 43.62 ± 1.44 | 0.00 ± 0.36 | 0.968^b^ |  | 43.39 ± 1.59 | 43.36 ± 1.61 | -0.03 ± 0.31 | **0.019^a*^** |
| Steep TK (D) | 44.73 ± 1.36 | 44.77 ± 1.36 | 0.05 ± 0.32 | 0.113^b^ |  | 44.34 ± 1.58 | 44.35 ± 1.60 | 0.01 ± 0.32 | 0.641^a^ |
| Mean TK (D) | 44.17 ± 1.36 | 44.19 ± 1.34 | 0.02 ± 0.27 | 0.344^b^ |  | 43.87 ± 1.56 | 43.85 ± 1.57 | -0.01 ± 0.25 | 0.385^b^ |
| TK-AK (D) | -0.03 ± 0.10 | -0.02 ± 0.11 | 0.01 ± 0.07 | 0.225^a^ |  | -0.01 ± 0.11 | -0.01 ± 0.11 | 0.00 ± 0.06 | 0.794^b^ |
| ACA (D) | 1.02 ± 0.73 | 1.04 ± 0.77 | 0.03 ± 0.35 | 0.394^b^ |  | 0.94 ± 0.63 | 0.95 ± 0.63 | 0.01 ± 0.35 | 0.473^a^ |
| PCA (D) | -0.32 ± 0.14 | -0.32 ± 0.16 | 0.00 ± 0.09 | 0.724^b^ |  | -0.32 ± 0.14 | -0.32 ± 0.14 | 0.00 ± 0.10 | 0.385^b^ |
| TCA (D) | 1.11 ± 0.73 | 1.16 ± 0.79 | 0.05 ± 0.40 | 0.177^a^ |  | 0.95 ± 0.63 | 0.98 ± 0.64 | 0.03 ± 0.38 | 0.052^a^ |
| CCT (mm) | 0.55 ± 0.03 | 0.55 ± 0.04 | 0.00 ± 0.01 | **0.043^b*^** |  | 0.54 ± 0.03 | 0.54 ± 0.03 | 0.00 ± 0.01 | 0.729^b^ |
| ACD (mm) | 3.01 ± 0.38 | 3.02 ± 0.40 | 0.01 ± 0.10 | 0.439^b^ |  | 3.17 ± 0.43 | 3.18 ± 0.44 | 0.00 ± 0.10 | 0.950^a^ |
| LT (mm) | 4.67 ± 0.50 | 4.68 ± 0.43 | 0.00 ± 0.34 | 0.906^a^ |  | 4.39 ± 0.45 | 4.37 ± 0.50 | -0.02 ± 0.36 | **0.031^a*^** |
| WTW(mm) | 11.73 ± 0.42 | 11.72 ± 0.48 | -0.01 ± 0.41 | 0.802^a^ |  | 11.80 ± 0.44 | 11.82 ± 0.44 | 0.01 ± 0.27 | 0.342^a^ |
| Angle alpha (mm) | 0.45 ± 0.21 | 0.48 ± 0.27 | 0.03 ± 0.31 | 0.624^a^ |  | 0.43 ± 0.22 | 0.44 ± 0.24 | 0.00 ± 0.21 | 0.987^a^ |
| Angle kappa (mm) | 0.24 ± 0.13 | 0.25 ± 0.14 | 0.01 ± 0.13 | 0.440^a^ |  | 0.26 ± 0.15 | 0.27 ± 0.15 | 0.00 ± 0.13 | 0.998^a^ |

Data are presented as mean ± standard deviation or as number (percent)

Statistically significant values (*p* < 0.05) are shown in asterisk (^*^)

^a^ Wilcoxon signed rank test

^b^ Paired t-test

AL, axial length; AK, anterior keratometry; D, diopter; PK, posterior keratometry; TK, total keratometry; ACA, anterior corneal astigmatism; PCA, posterior corneal astigmatism; TCA, total corneal astigmatism; CCT, central corneal thickness; ACD, anterior chamber depth; LT, lens thickness; WTW, white-to-white distance

**Supplementary Table 5.** Longitudinal change in ocular biometry measured by IOL Master 700 according to axial length.

| Parameter | **AL < 23 mm**  (N = 80) | | | |  | **23 mm ≤ AL < 25 mm**  (N = 241) | | | |  | **AL ≥ 25mm**  (N = 127) | | | |
| --- | --- | --- | --- | --- | --- | --- | --- | --- | --- | --- | --- | --- | --- | --- |
|  | Initial  measurement | Final  measurement | Difference  (Final- Initial) | *p*-value |  | Initial  measurement | Final  measurement | Difference  (Final- Initial) | *p*-value |  | Initial  measurement | Final  measurement | Difference  (Final- Initial) | *p*-value |
| Age (year) | 69.99 ± 8.85 | 71.92 ± 8.73 | 1.91 ± 0.79 | **<0.001^a*^** |  | 68.01 ± 10.42 | 70.00 ± 10.39 | 1.99 ± 0.80 | **<0.001^a*^** |  | 54.09 ± 14.69 | 55.97 ± 14.72 | 1.88 ± 0.70 | **<0.001^a*^** |
| Sex (male/female) | 11 (14%) / 69 (86%) | |  |  |  | 90 (37%) / 151 (63%) | |  |  |  | 60 (47%) / 67 (53%) | |  |  |
| Laterality (right/left) | 37 (46%) / 43 (54%) | |  |  |  | 117 (49%) / 124 (51%) | |  |  |  | 52 (41%) / 75 (59%) | |  |  |
| AL (mm) | 22.48 ± 0.42 | 22.51 ± 0.42 | 0.03 ± 0.05 | **<0.001^a*^** |  | 23.87 ± 0.55 | 23.89 ± 0.55 | 0.02 ± 0.05 | **<0.001^a*^** |  | 27.24 ± 1.80 | 27.31 ± 1.85 | 0.07 ± 0.16 | **<0.001^a*^** |
| Flat AK (D) | 45.01 ± 1.30 | 45.00 ± 1.30 | -0.01 ± 0.28 | 0.546^a^ |  | 43.39 ± 1.27 | 43.38 ± 1.30 | -0.01 ± 0.29 | 0.245^a^ |  | 42.69 ± 1.50 | 42.67 ± 1.50 | -0.01 ± 0.27 | 0.551^b^ |
| Steep AK (D) | 45.79 ± 1.34 | 45.79 ± 1.33 | 0.00 ± 0.31 | 0.897^b^ |  | 44.32 ± 1.28 | 44.33 ± 1.29 | 0.00 0.29 | 0.828^b^ |  | 43.81 ± 1.64 | 43.81 ± 1.65 | 0.00 ± 0.31 | 0.855^a^ |
| Mean AK (D) | 45.40 ± 1.31 | 45.40 ± 1.29 | 0.00 ± 0.23 | 0.901^b^ |  | 43.86 ± 1.23 | 43.85 ± 1.25 | 0.00 ± 0.23 | 0.791^b^ |  | 43.25 ± 1.53 | 43.24 ± 1.53 | -0.01 ± 0.25 | 0.738^b^ |
| Flat PK (D) | -5.99 ± 0.20 | -5.99 ± 0.20 | 0.00 ± 0.08 | 0.915^b^ |  | -5.76 ± 0.19 | -5.76 ± 0.19 | 0.00 ± 0.07 | 0.826^b^ |  | -5.65 ± 0.25 | -5.65 ± 0.24 | 0.00 ± 0.08 | 0.804^b^ |
| Steep PK (D) | -6.31 ± 0.22 | -6.29 ± 0.23 | 0.02 ± 0.09 | 0.281^a^ |  | -6.07 ± 0.22 | -6.06 ± 0.23 | 0.01 ± 0.08 | 0.328^b^ |  | -6.00 ± 0.28 | -6.00 ± 0.28 | 0.00 ± 0.08 | 0.567^b^ |
| Mean PK (D) | -6.15 ± 0.20 | -6.14 ± 0.21 | 0.01 ± 0.06 | 0.234^b^ |  | -5.91 ± 0.20 | -5.91 ± 0.20 | 0.00 ± 0.06 | 0.412^b^ |  | -5.82 ± 0.25 | -5.82 ± 0.25 | 0.00 ± 0.06 | 0.731^a^ |
| Flat TK (D) | 44.95 ± 1.34 | 44.93 ± 1.31 | -0.02 ± 0.33 | 0.511^b^ |  | 43.34 ± 1.29 | 43.32 ± 1.32 | -0.02 ± 0.34 | 0.097^a^ |  | 42.70 ± 1.51 | 42.69 ± 1.53 | -0.02 ± 0.30 | 0.515^b^ |
| Steep TK (D) | 45.77 ± 1.35 | 45.81 ± 1.34 | 0.04 ± 0.31 | 0.308^b^ |  | 44.34 ± 1.27 | 44.36 ± 1.28 | 0.02 ± 0.31 | 0.312^b^ |  | 43.79 ± 1.61 | 43.79 ± 1.61 | 0.00 ± 0.35 | 0.729^a^ |
| Mean TK (D) | 45.36 ± 1.32 | 45.37 ± 1.30 | 0.01 ± 0.25 | 0.848^b^ |  | 43.84 ± 1.23 | 43.84 ± 1.25 | 0.00 ± 0.25 | 0.938^b^ |  | 43.25 ± 1.52 | 43.24 ± 1.53 | -0.01 ± 0.28 | 0.662^b^ |
| TK-AK (D) | -0.04 ± 0.11 | -0.03 ± 0.11 | 0.01 ± 0.06 | 0.217^b^ |  | -0.02 ± 0.10 | -0.01 ± 0.10 | 0.00 ± 0.06 | 0.973^a^ |  | 0.00 ± 0.13 | -0.01 ± 0.13 | 0.00 ± 0.07 | 0.554^b^ |
| ACA (D) | 0.78 ± 0.42 | 0.79 ± 0.44 | 0.02 ± 0.38 | 0.717^b^ |  | 0.93 ± 0.66 | 0.95 ± 0.68 | 0.02 ± 0.36 | 0.488^b^ |  | 1.13 ± 0.72 | 1.14 ± 0.75 | 0.01 ± 0.32 | 0.619^b^ |
| PCA (D) | -0.32 ± 0.13 | -0.30 ± 0.12 | 0.02 ± 0.11 | 0.209^b^ |  | -0.31 ± 0.14 | -0.30 ± 0.15 | 0.00 ± 0.10 | 0.516^b^ |  | -0.35 ± 0.14 | -0.35 ± 0.15 | 0.00 ± 0.09 | 0.787^b^ |
| TCA (D) | 0.82 ± 0.47 | 0.88 ± 0.48 | 0.06 ± 0.41 | 0.159^a^ |  | 0.99 ± 0.69 | 1.04 ± 0.72 | 0.04 ± 0.40 | 0.099^b^ |  | 1.08 ± 0.70 | 1.10 ± 0.72 | 0.01 ± 0.34 | 0.664^b^ |
| CCT (mm) | 0.54 ± 0.03 | 0.54 ± 0.03 | 0.00 ± 0.01 | 0.176^b^ |  | 0.54 ± 0.03 | 0.54 ± 0.03 | 0.00 ± 0.01 | 0.597^b^ |  | 0.55 ± 0.04 | 0.55 ± 0.04 | 0.00 ± 0.01 | 0.432^b^ |
| ACD (mm) | 2.72 ± 0.37 | 2.73 ± 0.40 | 0.01 ± 0.10 | 0.832^a^ |  | 3.09 ± 0.34 | 3.10 ± 0.36 | 0.01 ± 0.08 | 0.095^a^ |  | 3.48 ± 0.32 | 3.46 ± 0.33 | -0.01 ± 0.11 | 0.102^a^ |
| LT (mm) | 4.72 ± 0.39 | 4.68 ± 0.45 | -0.04 ± 0.20 | 0.843^a^ |  | 4.51 ± 0.43 | 4.51 ± 0.40 | 0.00 ± 0.26 | 0.652^a^ |  | 4.20 ± 0.51 | 4.18 ± 0.59 | -0.02 ± 0.53 | **0.007^a*^** |
| WTW(mm) | 11.45 ± 0.37 | 11.49 ± 0.38 | 0.04 ± 0.30 | 0.264^a^ |  | 11.79 ± 0.39 | 11.80 ± 0.40 | 0.01 ± 0.30 | 0.731^a^ |  | 11.98 ± 0.43 | 11.97 ± 0.48 | -0.02 ± 0.35 | 0.776^a^ |
| Angle alpha (mm) | 0.51 ± 0.25 | 0.52 ± 0.24 | 0.01 ± 0.30 | 0.312^a^ |  | 0.46 ± 0.19 | 0.47 ± 0.23 | 0.01 ± 0.22 | 0.987^a^ |  | 0.34 ± 0.21 | 0.35 ± 0.27 | 0.01 ± 0.24 | 0.740^a^ |
| Angle kappa (mm) | 0.29 ± 0.15 | 0.28 ± 0.14 | -0.01 ± 0.13 | 0.652^a^ |  | 0.25 ± 0.14 | 0.27 ± 0.16 | 0.02 ± 0.13 | **0.022^a*^** |  | 0.26 ± 0.16 | 0.24 ± 0.14 | -0.02 ± 0.13 | **0.032^a*^** |

Data are presented as mean ± standard deviation or as number (percent)

Statistically significant values (*p* < 0.05) are shown in asterisk (^*^)

^a^ Wilcoxon signed rank test

^b^ Paired t-test

AL, axial length; AK, anterior keratometry; D, diopter; PK, posterior keratometry; TK, total keratometry; ACA, anterior corneal astigmatism; PCA, posterior corneal astigmatism; TCA, total corneal astigmatism; CCT, central corneal thickness; ACD, anterior chamber depth; LT, lens thickness; WTW, white-to-white distance

**Supplementary Table 6.** Longitudinal change in ocular biometry measured by IOL Master 700 according to degree of anterior corneal astigmatism.

| Parameter | **ACA ≥ 2 D**  (N = 32) | | | |  | **ACA < 2 D**  (N = 416) | | | |
| --- | --- | --- | --- | --- | --- | --- | --- | --- | --- |
|  | Initial  measurement | Final  measurement | Difference  (Final- Initial) | *p*-value |  | Initial  measurement | Final  measurement | Difference  (Final- Initial) | *p*-value |
| Age (year) | 57.36 ± 18.00 | 59.25 ± 18.07 | 1.89 ± 0.79 | **<0.001^a*^** |  | 64.96 ± 12.69 | 66.91 ± 12.68 | 1.95 ± 0.77 | **<0.001^b*^** |
| Sex (male/female) | 14 (44%) / 18 (56%) | |  |  |  | 90 (37%) / 151 (63%) | | 147 (35%) / 269 (65%) |  |
| Laterality (right/left) | 14 (44%) / 18 (56%) | |  |  |  | 117 (49%) / 124 (51%) | | 192 (46%) / 224 (54%) |  |
| AL (mm) | 25.92 ± 2.48 | 25.97 ± 2.54 | 0.05 ± 0.10 | **<0.001^b*^** |  | 24.47 ± 1.97 | 24.51 ± 2.00 | 0.04 ± 0.10 | **<0.001^b*^** |
| Flat AK (D) | 42.65 ± 1.53 | 42.67 ± 1.65 | 0.02 ± 0.29 | 0.679^a^ |  | 43.54 ± 1.53 | 43.53 ± 1.54 | -0.01 ± 0.28 | 0.090^b^ |
| Steep AK (D) | 45.28 ± 1.58 | 45.25 ± 1.57 | -0.03 ± 0.35 | 0.748^b^ |  | 44.38 ± 1.53 | 44.38 ± 1.54 | 0.01 ± 0.30 | 0.790^b^ |
| Mean AK (D) | 43.97 ± 1.53 | 43.96 ± 1.57 | -0.01 ± 0.25 | 0.881^a^ |  | 43.96 ± 1.51 | 43.96 ± 1.52 | 0.00 ± 0.23 | 0.691^a^ |
| Flat PK (D) | -5.69 ± 0.21 | -5.67 ± 0.24 | 0.02 ± 0.07 | 0.205^a^ |  | -5.77 ± 0.24 | -5.78 ± 0.24 | 0.00 ± 0.07 | 0.795^a^ |
| Steep PK (D) | -6.18 ± 0.26 | -6.17 ± 0.28 | 0.01 ± 0.07 | 0.580^a^ |  | -6.08 ± 0.26 | -6.08 ± 0.26 | 0.00 ± 0.08 | 0.493^b^ |
| Mean PK (D) | -5.93 ± 0.22 | -5.92 ± 0.24 | 0.01 ± 0.05 | 0.194^a^ |  | -5.93 ± 0.24 | -5.93 ± 0.24 | 0.00 ± 0.06 | 0.556^a^ |
| Flat TK (D) | 42.66 ± 1.59 | 42.70 ± 1.70 | 0.03 ± 0.30 | 0.507^a^ |  | 43.51 ± 1.54 | 43.48 ± 1.55 | -0.03 ± 0.33 | **0.013^b*^** |
| Steep TK (D) | 45.23 ± 1.56 | 45.20 ± 1.55 | -0.02 ± 0.41 | 0.774^b^ |  | 44.38 ± 1.52 | 44.40 ± 1.54 | 0.02 ± 0.31 | 0.263^b^ |
| Mean TK (D) | 43.95 ± 1.55 | 43.95 ± 1.57 | 0.00 ± 0.28 | 0.942^a^ |  | 43.94 ± 1.51 | 43.94 ± 1.52 | 0.00 ± 0.26 | 0.795^a^ |
| TK-AK (D) | -0.02 ± 0.09 | -0.01 ± 0.10 | 0.01 ± 0.06 | 0.285^a^ |  | -0.02 ± 0.11 | -0.01 ± 0.11 | 0.00 ± 0.06 | 0.680^a^ |
| ACA (D) | 2.63 ± 0.54 | 2.58 ± 0.73 | -0.05 ± 0.43 | 0.556^a^ |  | 0.83 ± 0.46 | 0.85 ± 0.48 | 0.02 ± 0.35 | 0.228^a^ |
| PCA (D) | -0.49 ± 0.18 | -0.50 ± 0.21 | -0.01 ± 0.10 | 0.634^a^ |  | -0.31 ± 0.13 | -0.30 ± 0.13 | 0.01 ± 0.10 | 0.261^a^ |
| TCA (D) | 2.56 ± 0.60 | 2.51 ± 0.83 | -0.06 ± 0.44 | 0.301^b^ |  | 0.87 ± 0.49 | 0.91 ± 0.52 | 0.04 ± 0.38 | **0.006^b*^** |
| CCT (mm) | 0.54 ± 0.03 | 0.54 ± 0.04 | 0.00 ± 0.01 | 0.059^a^ |  | 0.54 ± 0.03 | 0.54 ± 0.03 | 0.00 ± 0.01 | 0.308^a^ |
| ACD (mm) | 3.21 ± 0.38 | 3.22 ± 0.38 | 0.01 ± 0.10 | 0.761^a^ |  | 3.13 ± 0.43 | 3.13 ± 0.44 | 0.00 ± 0.10 | 0.747^b^ |
| LT (mm) | 4.36 ± 0.52 | 4.39 ± 0.51 | 0.03 ± 0.08 | 0.072^a^ |  | 4.47 ± 0.48 | 4.45 ± 0.50 | -0.02 ± 0.37 | 0.164^b^ |
| WTW(mm) | 11.80 ± 0.47 | 11.79 ± 0.51 | -0.02 ± 0.14 | 0.575^a^ |  | 11.78 ± 0.43 | 11.79 ± 0.44 | 0.01 ± 0.32 | 0.285^b^ |
| Angle alpha (mm) | 0.38 ± 0.19 | 0.37 ± 0.23 | -0.01 ± 0.17 | 0.760^a^ |  | 0.44 ± 0.22 | 0.45 ± 0.25 | 0.01 ± 0.24 | 0.584^b^ |
| Angle kappa (mm) | 0.27 ± 0.13 | 0.28 ± 0.14 | 0.02 ± 0.13 | 0.970^b^ |  | 0.26 ± 0.15 | 0.26 ± 0.15 | 0.00 ± 0.13 | 0.691^b^ |

Data are presented as mean ± standard deviation or as number (percent)

Statistically significant values (*p* < 0.05) are shown in asterisk (^*^)

^a^ Paired t-test

^b^ Wilcoxon signed rank test

ACA, anterior corneal astigmatism; D, diopter; AL, axial length; AK, anterior keratometry; PK, posterior keratometry; TK, total keratometry; PCA, posterior corneal astigmatism; TCA, total corneal astigmatism; CCT, central corneal thickness; ACD, anterior chamber depth; LT, lens thickness; WTW, white-to-white distance

**Supplementary Table 7.** Longitudinal change in ocular biometry measured by IOL Master 700 according to measurement intervals.

| Parameter | **1 to 2 Years Apart**  (N = 282) | | | |  | **≥ 2 Years Apart**  (N = 166) | | | |
| --- | --- | --- | --- | --- | --- | --- | --- | --- | --- |
|  | Initial  measurement | Final  measurement | Difference  (Final- Initial) | *p*-value |  | Initial  measurement | Final  measurement | Difference  (Final- Initial) | *p*-value |
| Age (year) | 64.09 ± 13.98 | 65.54 ± 13.95 | 1.45 ± 0.31 | **<0.001^a*^** |  | 64.98 ± 11.96 | 67.76 ± 11.90 | 2.79 ± 0.57 | **<0.001^a*^** |
| Sex (male/female) | 104 (37%) / 178 (63%) | |  |  |  | 86 (34%) / 164 (66%) | | 57 (34%) / 109 (66%) |  |
| Laterality (right/left) | 131 (46%) / 151 (54%) | |  |  |  | 113 (45%) / 137 (55%) | | 75 (45%) / 91 (55%) |  |
| AL (mm) | 24.67 ± 2.09 | 24.70 ± 2.11 | 0.03 ± 0.06 | **<0.001^a*^** |  | 24.42 ± 1.97 | 24.47 ± 2.01 | 0.06 ± 0.13 | **<0.001^a*^** |
| Flat AK (D) | 43.35 ± 1.56 | 43.34 ± 1.58 | -0.02 ± 0.27 | 0.114^a^ |  | 43.69 ± 1.50 | 43.69 ± 1.52 | 0.00 ± 0.31 | 0.675^a^ |
| Steep AK (D) | 44.33 ± 1.55 | 44.33 ± 1.55 | 0.00 ± 0.29 | 0.651^a^ |  | 44.63 ± 1.53 | 44.64 ± 1.54 | 0.01 ± 0.33 | 0.594^b^ |
| Mean AK (D) | 43.84 ± 1.52 | 43.83 ± 1.53 | -0.01 ± 0.22 | 0.408^b^ |  | 44.16 ± 1.48 | 44.17 ± 1.49 | 0.01 ± 0.26 | 0.777^b^ |
| Flat PK (D) | -5.75 ± 0.24 | -5.75 ± 0.24 | 0.00 ± 0.07 | 0.966^b^ |  | -5.80 ± 0.24 | -5.80 ± 0.23 | 0.00 ± 0.08 | 0.963^b^ |
| Steep PK (D) | -6.07 ± 0.26 | -6.07 ± 0.26 | 0.00 ± 0.07 | 0.510^a^ |  | -6.13 ± 0.26 | -6.12 ± 0.26 | 0.01 ± 0.10 | 0.063^a^ |
| Mean PK (D) | -5.91 ± 0.24 | -5.91 ± 0.24 | 0.00 ± 0.06 | 0.990^b^ |  | -5.97 ± 0.23 | -5.96 ± 0.24 | 0.01 ± 0.07 | 0.215^b^ |
| Flat TK (D) | 43.33 ± 1.57 | 43.30 ± 1.59 | -0.03 ± 0.31 | **0.032^a*^** |  | 43.65 ± 1.51 | 43.64 ± 1.52 | -0.01 ± 0.35 | 0.432^a^ |
| Steep TK (D) | 44.33 ± 1.54 | 44.34 ± 1.55 | 0 01 ± 0.31 | 0.903^a^ |  | 44.62 ± 1.52 | 44.65 ± 1.54 | 0.03 ± 0.34 | 0.209^b^ |
| Mean TK (D) | 43.83 ± 1.52 | 43.83 ± 1.53 | -0.01 ± 0.24 | 0.399^b^ |  | 44.14 ± 1.48 | 44.15 ± 1.49 | 0.01 ± 0.28 | 0.546^b^ |
| TK-AK (D) | -0.01 ± 0.11 | -0.01 ± 0.11 | 0.00 ± 0.06 | 0.677^b^ |  | -0.02 ± 0.10 | -0.02 ± 0.11 | 0.01 ± 0.07 | 0.159^b^ |
| ACA (D) | 0.98 ± 0.68 | 0.99 ± 0.69 | 0.02 ± 0.34 | 0.280^a^ |  | 0.93 ± 0.61 | 0.95 ± 0.63 | 0.02 ± 0.38 | 0.592^b^ |
| PCA (D) | -0.32 ± 0.14 | -0.32 ± 0.15 | 0.00 ± 0.09 | 0.935^b^ |  | -0.33 ± 0.15 | -0.32 ± 0.14 | 0.01 ± 0.11 | 0.146^b^ |
| TCA (D) | 1.00 ± 0.69 | 1.04 ± 0.70 | 0.04 ± 0.38 | 0.111^b^ |  | 0.97 ± 0.61 | 1.01 ± 0.65 | 0.04 ± 0.40 | 0.220^a^ |
| CCT (mm) | 0.54 ± 0.03 | 0.54 ± 0.03 | 0.00 ± 0.01 | 0.662^a^ |  | 0.54 ± 0.03 | 0.54 ± 0.03 | 0.00 ± 0.01 | 0.160^b^ |
| ACD (mm) | 3.16 ± 0.43 | 3.16 ± 0.44 | 0.00 ± 0.09 | 0.726^a^ |  | 3.09 ± 0.41 | 3.10 ± 0.43 | 0.01 ± 0.11 | 0.324^a^ |
| LT (mm) | 4.42 ± 0.50 | 4.42 ± 0.49 | 0.00 ± 0.37 | 0.565^a^ |  | 4.53 ± 0.43 | 4.49 ± 0.52 | -0.03 ± 0.32 | 0.070^a^ |
| WTW(mm) | 11.83 ± 0.45 | 11.82 ± 0.44 | -0.01 ± 0.29 | 0.791^a^ |  | 11.71 ± 0.40 | 11.75 ± 0.47 | 0.04 ± 0.35 | 0.055^a^ |
| Angle alpha (mm) | 0.45 ± 0.23 | 0.45 ± 0.26 | 0.00 ± 0.24 | 0.874^a^ |  | 0.42 ± 0.19 | 0.43 ± 0.23 | 0.02 ± 0.23 | 0.751^a^ |
| Angle kappa (mm) | 0.26 ± 0.15 | 0.26 ± 0.14 | 0.00 ± 0.13 | 0.913^a^ |  | 0.25 ± 0.14 | 0.26 ± 0.16 | 0.01 ± 0.13 | 0.409^a^ |

Data are presented as mean ± standard deviation or as number (percent)

Statistically significant values (*p* < 0.05) are shown in asterisk (^*^)

^a^ Wilcoxon signed rank test

^b^ Paired t-test

AL, axial length; AK, anterior keratometry; D, diopter; PK, posterior keratometry; TK, total keratometry; ACA, anterior corneal astigmatism; PCA, posterior corneal astigmatism; TCA, total corneal astigmatism; CCT, central corneal thickness; ACD, anterior chamber depth; LT, lens thickness; WTW, white-to-white distance

**Supplementary Table 8.** Longitudinal change in ocular biometry measured by IOL Master 700 in 119 eyes that later underwent cataract surgery.

| Parameter | Initial  measurement | Final  measurement | Difference  (Final-Initial) | *p*-value |  |
| --- | --- | --- | --- | --- | --- |
| Age (year) | 66.76 ± 12.12 | 68.72 ± 12.09 | 1.97 ± 0.73 | **<0.001^a*^** |  |
| Sex (male/female) | 48 (40%) / 71 (60%) | |  |  |  |
| Laterality (right/left) | 53 (45%) / 66 (55%) | |  |  |  |
| AL (mm) | 24.34 ± 1.71 | 24.37 ± 1.72 | 0.02 ± 0.05 | **<0.001^a*^** |  |
| Flat AK (D) | 43.58 ± 1.48 | 43.56 ± 1.50 | -0.02 ± 0.31 | 0.105^a^ |  |
| Steep AK (D) | 44.42 ± 1.48 | 44.41 ± 1.46 | 0.00 ± 0.29 | 0.546^a^ |  |
| Mean AK (D) | 44.00 ± 1.46 | 43.99 ± 1.45 | -0.01 ± 0.24 | 0.263^a^ |  |
| Flat PK (D) | -5.79 ± 0.24 | -5.78 ± 0.23 | 0.01 ± 0.08 | 0.080^b^ |  |
| Steep PK (D) | -6.09 ± 0.27 | -6.08 ± 0.27 | 0.01 ± 0.08 | 0.102^b^ |  |
| Mean PK (D) | -5.94 ± 0.25 | -5.93 ± 0.24 | 0.01 ± 0.06 | **0.029^b*^** |  |
| Flat TK (D) | 43.52 ± 1.47 | 43.50 ± 1.49 | -0.01 ± 0.37 | 0.136^a^ |  |
| Steep TK (D) | 44.43 ± 1.47 | 44.45 ± 1.45 | 0.02 ± 0.31 | 0.961^a^ |  |
| Mean TK (D) | 43.97 ± 1.44 | 43.97 ± 1.44 | 0.00 ± 0.27 | 0.615^a^ |  |
| TK-AK (D) | -0.02 ± 0.11 | -0.01 ± 0.11 | 0.01 ± 0.07 | 0.061^b^ |  |
| ACA (D) | 0.84 ± 0.51 | 0.85 ± 0.54 | 0.02 ± 0.38 | 0.658^b^ |  |
| PCA (D) | -0.30 ± 0.12 | -0.30 ± 0.13 | 0.00 ± 0.10 | 0.995^b^ |  |
| TCA (D) | 0.91 ± 0.57 | 0.94 ± 0.61 | 0.03 ± 0.42 | 0.431^b^ |  |
| CCT (mm) | 0.54 ± 0.03 | 0.54 ± 0.03 | 0.00 ± 0.01 | 0.077^b^ |  |
| ACD (mm) | 3.19 ± 0.42 | 3.20 ± 0.42 | 0.01 ± 0.10 | 0.486^a^ |  |
| LT (mm) | 4.47 ± 0.43 | 4.47 ± 0.46 | 0.00 ± 0.15 | 0.413^a^ |  |
| WTW(mm) | 11.82 ± 0.41 | 11.78 ± 0.49 | -0.04 ± 0.36 | 0.475^a^ |  |
| Angle alpha (mm) | 0.43 ± 0.20 | 0.45 ± 0.24 | 0.02 ± 0.25 | 0.550^a^ |  |
| Angle kappa (mm) | 0.25 ± 0.14 | 0.24 ± 0.16 | 0.00 ± 0.11 | 0.382^a^ |  |

Data are presented as mean ± standard deviation or as number (percent)

Statistically significant values (*p* < 0.05) are shown in asterisk (^*^)

^a^ Wilcoxon signed rank test

^b^ Paired t-test

AL, axial length; AK, anterior keratometry; D, diopter; PK, posterior keratometry; TK, total keratometry; ACA, anterior corneal astigmatism; PCA, posterior corneal astigmatism; TCA, total corneal astigmatism; CCT, central corneal thickness; ACD, anterior chamber depth; LT, lens thickness; WTW, white-to-white distance
